# Structural Insights into the Interactions of Candidal Enolase with Human Vitronectin, Fibronectin and Plasminogen

**DOI:** 10.3390/ijms21217843

**Published:** 2020-10-22

**Authors:** Dorota Satala, Grzegorz Satala, Justyna Karkowska-Kuleta, Michal Bukowski, Anna Kluza, Maria Rapala-Kozik, Andrzej Kozik

**Affiliations:** 1 Department of Analytical Biochemistry, Faculty of Biochemistry, Biophysics and Biotechnology, Jagiellonian University in Krakow, 30-387 Kraków, Poland; dorota.satala@uj.edu.pl (D.S.); m.bukowski@uj.edu.pl (M.B.); 2 Department of Medicinal Chemistry, Maj Institute of Pharmacology, Polish Academy of Sciences, 31-343 Kraków, Poland; satala@if-pan.krakow.pl; 3 Department of Comparative Biochemistry and Bioanalytics, Faculty of Biochemistry, Biophysics and Biotechnology, Jagiellonian University in Krakow, 30-387 Kraków, Poland; justyna.karkowska@uj.edu.pl (J.K.-K.); nckluza@cyf-kr.edu.pl (A.K.); maria.rapala-kozik@uj.edu.pl (M.R.-K.)

**Keywords:** non-albicans Candida species, enolase, moonlighting protein, vitronectin, fibronectin, plasminogen

## Abstract

Significant amounts of enolase—a cytosolic enzyme involved in the glycolysis pathway—are exposed on the cell surface of Candida yeast. It has been hypothesized that this exposed enolase form contributes to infection-related phenomena such as fungal adhesion to human tissues, and the activation of fibrinolysis and extracellular matrix degradation. The aim of the present study was to characterize, in structural terms, the protein-protein interactions underlying these moonlighting functions of enolase. The tight binding of human vitronectin, fibronectin and plasminogen by purified *C. albicans* and *C. tropicalis* enolases was quantitatively analyzed by surface plasmon resonance measurements, and the dissociation constants of the formed complexes were determined to be in the 10^−7^–10^−8^ M range. In contrast, the binding of human proteins by the *S.* *cerevisiae* enzyme was much weaker. The chemical cross-linking method was used to map the sites on enolase molecules that come into direct contact with human proteins. An internal motif _235_DKAGYKGKVGIAMDVASSEFYKDGK_259_ in *C. albicans* enolase was suggested to contribute to the binding of all three human proteins tested. Models for these interactions were developed and revealed the sites on the enolase molecule that bind human proteins, extensively overlap for these ligands, and are well-separated from the catalytic activity center.

## 1. Introduction

Enolase (2-phospho-D-glycerate hydrolase, EC 4.2.1.11) catalyzes the interconversion between 2-phosphoglycerate and phosphoenolpyruvate that occurs during the glycolysis (forward reaction) and gluconeogenesis (reverse reaction) pathways. Aside from this fundamental and evolutionally conserved function however, enolase is highly multifunctional (for reviews see: [1,2,3]). Since the first reports of “gene sharing” by enolase and τ-crystallin—a structural protein of the eye lens [4,5], numerous further studies have assigned enolase a status of one of the best characterized of the “moonlighting proteins”—single polypeptide chains that perform two or more fundamentally different and unrelated functions which are not due to gene fusion, alternative mRNA splicing, proteolytic generation of different protein variants or promiscuous enzyme activity [6]. These additional functions occur via a priori unexpected interactions with non-canonical molecular targets and sometimes at an “unauthorized” subcellular or extracellular location that is not consistent with the classical rules of protein sorting [7]. Enolase is primarily an intracellular (cytosolic) protein. In *Escherichia coli* cells, it moonlights as a component of the RNA degradosome [8,9]. In *Saccharomyces cerevisiae* yeast it serves as a heat shock protein, Hsp48 [10], and, in a complex with some other glycolytic enzymes, sticks to the outer mitochondrial membrane, thereby mediating the transport of aminoacyl-tRNAs to the mitochondrial matrix to facilitate intra-mitochondrial protein synthesis [11,12]. In the cytosol of mammalian cells enolase contributes to the regulation of cell morphology via interactions with tubulin and microtubules [13], binds cholesterol esters thus decreasing the cellular cholesterol level [14,15], and acts as a hypoxic stress protein, HAP47 [16,17]. Albeit lacking typical signal peptides, enolase also appears in the nucleus where it functions as a *c-myc* promoter-binding protein (MCB-1) to regulate the transcription of the *c-myc* proto-oncogene [18,19], and on various human cell surfaces where it can act as an autoantigen [20], or a receptor for plasminogen where it modulates pericellular fibrinolytic activity [1], with further pathophysiological consequences. Notably, enolase and an array of other moonlighting proteins are exposed on the surface of pathogenic bacteria and some eukaryotic pathogens, and contribute to their virulence, primarily via adherence to the host cells and proteins, or through interference with host immune mechanisms (for reviews see: [7,21,22,23,24]).

Recent proteomic studies have repeatedly demonstrated the presence of enolase at the cell surface of yeast-like fungi from the Candida genus [25,26,27,28], a major group of fungal pathogens in humans. Commensally inhabiting the skin and mucosal membranes of healthy individuals, these fungi can cause severe infections in hospitalized patients with impaired immune mechanisms [29]. Although *Candida albicans* is still the main fungal species isolated from the bloodstream, the incidence of infections caused by species other than *C. albicans*, which are an increasingly emerging cause of systemic and poorly curable candidiases, has been growing in recent decades [30,31]. One of these other pathogenic species is *C. tropicalis*, which is currently considered to be the second or third causative agent of fungal diseases, depending on geographical location, accounting for almost 31% of infections in Asia, 17% in the USA, and 15–21% in Latin America [32]. In several prior studies, malignancies, mainly in the hematopoietic system, were indicated as the dominant factor increasing the risk of infection with this species [33,34].

Several lines of evidence have clearly confirmed an important role of enolase in candidal virulence. For example, enolase was reported as the predominant antigen detected during *C. albicans*-dependent infections [35]. Moreover, a *C. albicans* enolase-null mutant, although capable of surviving and growing in media with a non-fermentable carbon source, exhibited increased susceptibility to certain antifungal drugs, was defective in hypha formation, and was avirulent in mice [36]. Owing to an additional transglutaminase activity, enolase takes part in the maintenance of cell wall integrity, in the morphological transition of fungi, and in the protection against osmotic stress [37]. This enzyme was also shown to be an important factor in the process of fungal adhesion to biomaterials such as polyvinylchloride and silicone, which are commonly used in medical devices including catheters, valves or orthopedic prostheses [38]. Like in other pathogenic microbes, multiple moonlighting proteins that are present on candidal cell surfaces have been primarily anticipated to contribute to the adhesion of the pathogen to the host tissues. This phenomenon is critical for the colonization and infection of the host, and these proteins, loosely associated with the fungal cell wall, probably act collectively [7]. They therefore complement the basic role of the major dedicated adhesins that are covalently anchored to the cell wall and are surface-displayed in a tightly controlled fashion in a response to signals from the host environment [39]. Many studies have pointed to enolase as a main moonlighting adhesin at the candidal cell surface [40,41,42], although the relative contribution of this protein to the entire phenomenon of candidal adhesion to the host can vary depending on the infection stage and context. In some reports, the role of enolase in fungus-host adhesion was suggested to be critical. A prior study reported the suppressive effects of anti-enolase antibodies and recombinant enolase on *C. albicans* adhesion to intestinal mucosa, which was found to decrease by 48–70% [40]. Proteins reported to be targets for candidal enolase in the human host have included the cadherins on epithelial and endothelial cells [40], to which the fungal pathogen adheres at the initial stages of host colonization [43], and during the later dissemination of the pathogen within the host organism [36,44], respectively. Additional target proteins include those of the basal membrane such as laminin (LAM), and extracellular matrix (ECM) components such as fibronectin (FN) or vitronectin (VTR) [45]. These structures become exposed after damage to the tight protective cell layers, thus providing additional points for pathogen attachment.

Another area in which the interactions of candidal moonlighting proteins, including enolase, with host proteins have been confirmed to contribute to fugal pathogenicity is the interference with the host homeostatic/hemostatic systems based on proteolytic cascades such as the fibrinolysis, complement and kallikrein-kinin systems (for a review see: [7]). The best studied example is the binding of human plasminogen (HPG), a phenomenon that has been reported to occur universally on the surfaces of many types of prokaryotic and eukaryotic cells [1]. Binding of HPG and its active form, plasmin, to the surface of the yeast allows the pathogen to participate in the process of clot fibrinolysis, enabling the release of pathogens trapped inside. Furthermore, the proteolytic activity of plasmin can also activate collagenases and mediators involved in the activation of the complement system, thereby contributing to the degradation of components of the human ECM [46,47] and facilitating the penetration of surrounding tissues [48,49]. Enolase is one of multiple HPG-binding moonlighting proteins found at the cell surface of *C. albicans* [48,50] and other medically important Candida species [51].

The current state of knowledge regarding moonlighting proteins in various taxa, including candidal enolase, does not include an extensive characterization of the structural factors that determine the tight interactions of these proteins with host proteinaceous targets. Such studies would add considerably to the discussion on how a priori unexpected moonlighting functions could have arisen during evolution through the adaptation of previously unused molecule surfaces as new binding sites for diverse ligands, and also without any changes to previous structural determinants of the main functions of the protein in question [52]. As long as this alternative function did not disrupt the core enzymatic function, these multifunctional proteins could have persisted during the course of evolution [23]. It seems that very small changes in a protein’s covalent structure can change its biological function [53]. These changes can be unique for a given protein and not necessarily conserved among its homologues [54]. The likely types of such changes would include, for example, posttranslational modifications, such as those reported for mammalian enolases [1]. An interesting issue, particularly important for candidal cytosolic proteins that unexpectedly appear on the cell surface, regards possible structural differences between these two forms.

Some of these issues are addressed in our present study, in which we aimed to conduct a structural characterization of the interactions between candidal enolase and the host proteins that have been repeatedly reported to be potential enolase ligands i.e., VTR, FN and HPG. The cytosolic and cell wall-associated enolase forms of two candidal species: *C. albicans* and *C. tropicalis* were compared in terms of their human protein-binding profiles. The complexes formed were mapped by chemical cross-linking and visualized by molecular modelling. For HPG, some reference data on its binding to enolase from other organisms are available [55,56,57], but no structural characteristics of ECM protein-enolase interactions have been reported previously.

## 2. Results

### 2.1. Soluble Purified Candidal Enolases and Anti-Enolase Antibodies Inhibit the Adsorption of Human VTR, FN and HPG to Fungal Cells

We obtained several forms of candidal enolase i.e., purified from the *C. albicans* cell wall and cytosol, purified from the *C. tropicalis* cytosol, and recombinant *C. albicans* proteins produced in *E. coli* (both wild-type and specifically designed mutant forms). The purified proteins were analyzed by sodium dodecyl sulphate-polyacrylamide gel electrophoresis (SDS-PAGE) (Figure S1 in the Supplementary material), unequivocally identified by liquid chromatography-coupled tandem mass spectrometry (LC-MS/MS), and confirmed to be non-glycosylated via carbohydrate-specific in-gel staining. The specific enzymatic activities of the purified enolases were comparable (ca. 100 units/mg protein).

**Figure 1:**
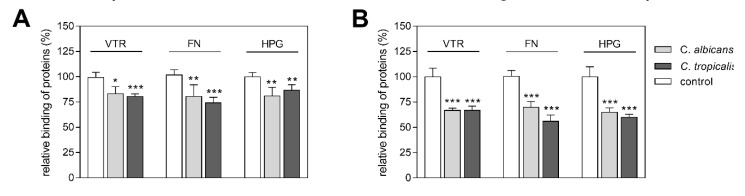
Effects of *C. albicans* and *C. tropicalis* purified enolases (**A**) or anti-enolase antibody (**B**) on the binding of biotin-labeled human proteins—vitronectin (VTR-Bt), fibronectin (FN-Bt), plasminogen (HPG-Bt)—to Candida spp. cells. Candida spp. hyphal or pseudohyphal forms, settled in the wells of a MaxiSorp microplate (1 × 10^6^ cells per well), were (**A**) incubated with mixtures of 40 µL of VTR-Bt, FN-Bt or HPG-Bt (at the final amount of 2 pmoles) with 10 µL of purified Candida spp. enolases (final amount of 24 pmoles); or (**B**) pre-incubated with the fungal anti-enolase antibodies (1 µg/µL and then treated with 50 µL of VTR-Bt, FN-Bt or HPG-Bt (at a final concentration of 250 nM). After washing off unbound protein, the amount of bound human protein was determined using the streptavidin-horseradish peroxidase/tetramethylbenzidine SA-HRP/TMB system. Bars represent the mean values from three determinations with standard deviations. Statistical significance levels against the 100% controls were determined using a one-way ANOVA test: * *p* < 0.05, ** *p* < 0.01 and *** *p* < 0.001.

First, to test the hypothesis that enolase exposed at the candidal cell surface contributes significantly to the phenomenon of human protein binding to the whole candidal cell, we analyzed the inhibitory effects of soluble fungal enolases and anti-enolase antibodies on the adsorption of VTR, FN and HPG to *C. albicans* and *C. tropicalis* hyphae/pseudohyphae (Figure 1). The results indicated that the fungal cell-binding of human proteins decreased by 20–25% and 30–40%, respectively, in the presence of competitors. This initial experiment provided a good biological rationale for subsequent structural analyses of the enolase interactions with the human host proteins under analysis.

### 2.2. The Equivalent Human Protein-Binding Abilities of C. albicans Cell Surface-Exposed and Cytosolic Enolases 

**Figure 2:**
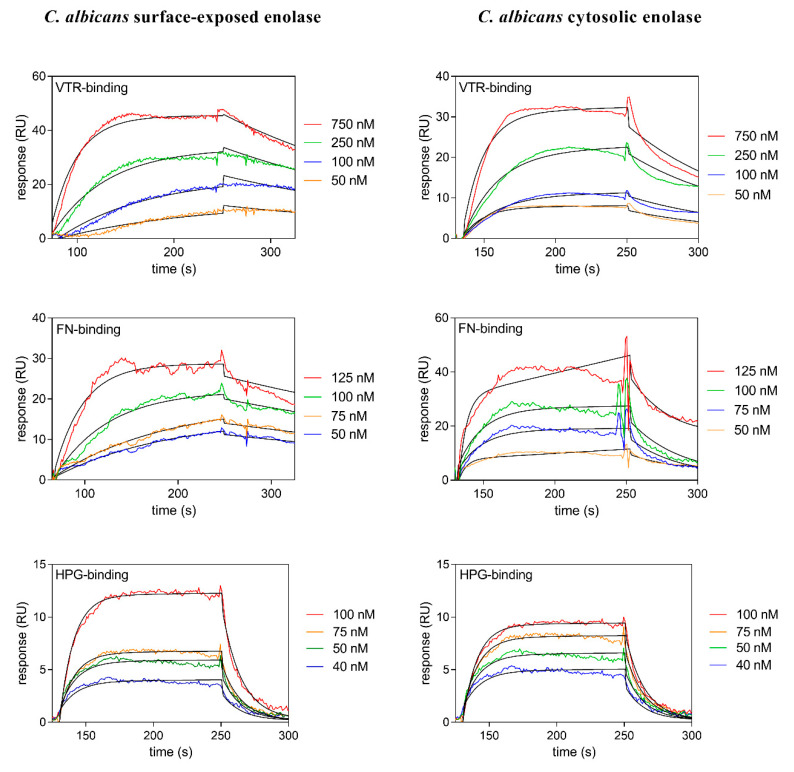
Surface plasmon resonance (SPR) analysis of the interactions between vitronectin (VTR), fibronectin (FN) or human plasminogen (HPG) and *C. albicans* enolase isolated from the cell surface or cytosol. Sensograms were obtained after the injection of human proteins (analyte concentrations were within a 40–750 nM range) over a CM5 chip containing immobilized enolase at a flow rate of 30 µL/min. A Langmuir 1:1 binding model with a baseline drift and Rmax locally was well fitted to the sensograms, as shown by the black lines. These data were collected from three independent experiments.

Two methods were utilized to compare the binding of the human VTR, FN and HPG proteins by both the cell surface-exposed and cytosolic forms of *C. albicans* enolase. The overall binding levels were first semi-quantitatively analyzed using a microplate ligand-binding assay, based on the interaction of biotin-labeled enolase with polystyrene-immobilized human protein (Figure S4 in the Supplementary material). Second, the advanced method of surface plasmon resonance (SPR) measurements was used to characterize the on-flow binding of soluble human protein with biocompatible (dextran-based) chip-immobilized enolase. The latter method generates quantitative binding parameters i.e., the kinetic association and dissociation rate constants, *k_a_* and *k_d_*, and the equilibrium dissociation constant *K_D_* (*K_D_ = k_d_*/*k_a_*). Moreover, it seems to be a better model for phenomenon studied as the enolase is immobilized in a polysaccharide environment of the candidal cell wall and is interacting with possibly soluble human proteins. The sensograms for the interactions within particular enolase-human protein pairs, analyzed in this series of experiments, are shown in Figure 2. The binding parameters obtained from all SPR analyzes performed throughout this entire study are presented in Table 1. 

Neither of these two methods revealed any noticeable differences between the capability of *C. albicans* surface-exposed and cytosolic enolases to interact with the human proteins being tested. Hence, most of the subsequent experiments were performed on cytosolic enolase, under the assumption that host proteins will interact with candidal enolases regardless of their origin. 

### 2.3. C. tropicalis Enolase Binds VTR, FN and HPG with Kinetic and Equilibrium Parameters that Are Comparable to Those of C. albicans Enolase, but Much Stronger than the S. cerevisiae Enolase

**Figure 3:**
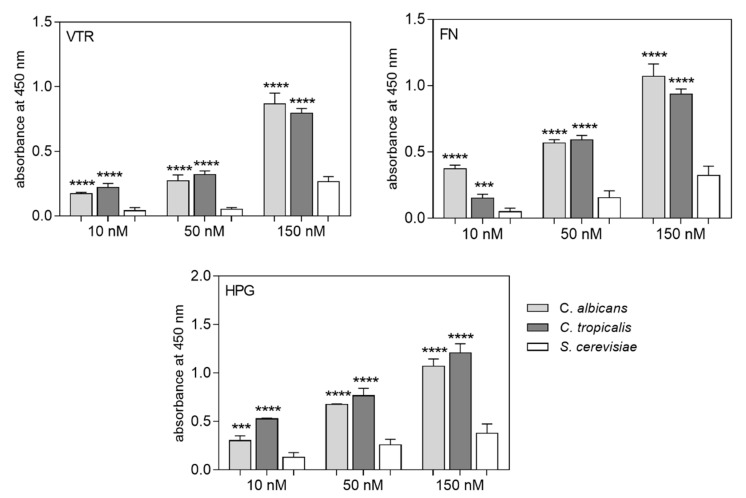
Binding of biotin-labeled fungal enolases to microplate-immobilized VTR, FN and HPG. Microplate wells coated with 3 pmoles of VTR, FN or HPG were filled with 50 µL of biotin-labeled fungal enolases at increasing concentrations. The amount of bound protein was determined with the SA-HRP/TMB system. The wells without human protein, which were surface-blocked with 3% bovine serum albumin (BSA), served as a control, and the values obtained for these samples were subtracted from the total binding measurements. The bars represent the mean values ± standard deviation (3 determinations). The levels of statistical significance were determined with one-way ANOVA in comparison to the signal obtained for S. cerevisiae enolase; *** *p* < 0.001 or **** *p* < 0.0001.

Using the microplate ligand-binding assay, we found that the cytosolic enolases isolated from *C. albicans* and *C. tropicalis* bound to VTR, FN and HPG to a comparable degree (Figure 3). In contrast to the candidal enolases however, the non-pathogenic yeast *S. cerevisiae* enolase showed a several-fold lower binding affinity for these human proteins. Thus, we surmised that substantial structural differences would likely exist between the Candida and Saccharomyces enolases at the regions of these molecules that make contact with human host factors. 

**Figure 4:**
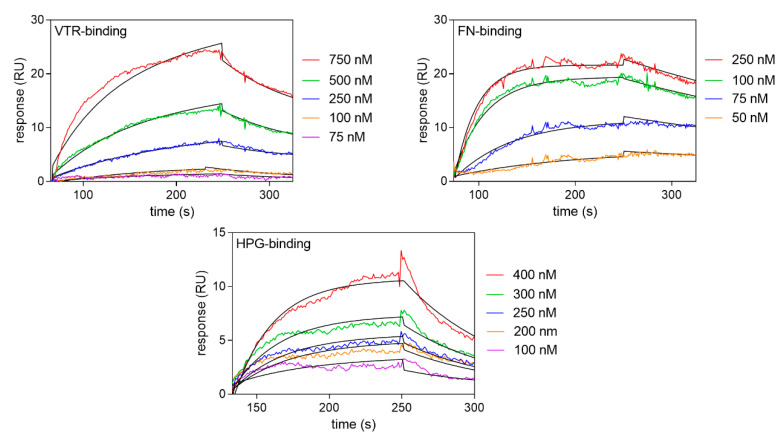
SPR analysis of the interactions between *C. tropicalis* enolase and VTR, FN and HPG. Sensograms were obtained after the injection of the human proteins (analyte concentrations were within a 75–750 nM range) over a CM5 chip containing immobilized enolase at a flow rate of 30 μL/min. A Langmuir 1:1 binding model with a baseline drift and Rmax locally was well fitted to the sensograms, as shown by the black lines. These data were collected from three independent experiments.

The interactions of *C. tropicalis* enolase with FN, VTR and HPG were additionally analyzed by SPR measurements. Representative sensograms from these experiments are presented in Figure 4, and the determined binding parameters are listed in Table 1 together with those for *C. albicans* enolases. For all of the candidal enolases and human proteins studied, the binding parameters—e.g., the *K_D_* constants ranging from 10^−7^ to 10^−8^ M—were comparable and indicative of quite strong protein-protein interactions. Interestingly however, the SPR method indicated a very poor, if any, interaction of *S. cerevisiae* enolase with these three human proteins (data not shown).

### 2.4. The Binding of VTR, FN and HPG to the Candidal Enolases is Partially Competitive and Does Not Affect Their Catalytic Activity 

It was clearly demonstrated in our current experiments that the candidal enolases, after forming a complex with VTR, FN or HPG, retained their enzymatic activity (a representative result is presented in Figure S5 in the Supplementary Materials). This finding suggested that the sites of *C. albicans* and *C. tropicalis* enolase molecules that directly interact with the human proteins are at an appreciable distance from the enolase active center. 

To initially assess for possible competition between these human proteins for binding to candidal enolases, that would likely arise if there was any overlap between the binding sites on the enzyme surface, we performed displacement tests in which biotinylated enolase and a molar excess of one human protein (unlabeled) were added together to microplates containing an immobilized second human protein. The total binding levels of the biotinylated enolase were determined and compared to those occurring in the absence of the soluble human protein. A representative result obtained for HPG (soluble) and VTR (immobilized) is presented in Figure S6 in the Supplementary Material. The presence of HPG in the solution reduced the binding of the biotinylated *C. albicans* enolase to immobilized VTR by about 50%. The addition of magnesium ions, that are required for the enzymatic activity of enolase, had no effects in these experiments.

### 2.5. Chemical Cross-Linking to Map the Candidal Enolase Sequence Fragments Involved in the Interactions with Human Host Proteins

To characterize the interactions of enolase with human proteins in more detail, we applied a chemical cross-linking method using a heterobifunctional, photoreactive reagent, sulfosuccinimidyl 2-([4,4′-azipentanamido]ethyl)-1,3′-dithiopropionate (sulfo-SDAD). In the first step, sulfo-SDAD was attached in the dark to amino residues located on the human proteins. After subsequent incubation with enolase, a covalent linkage of the enolase-human protein pairs occurred under UV light, with the cross-linking agent linking to the nearest fragment of the enolase. After cleaving the disulfide bridge in the sulfo-SDAD reagent, the proteins were digested with trypsin and the resulting peptides were analyzed by LC-MS/MS. Using a Mass Matrix server, peptides with a mass shift corresponding to the mass of the sulfo-SDAD fragment were searched for. The obtained results are summarized in Table 2 and the complete data set is shown in the Supplementary Materials (Table S1). This approach allowed us to identify the reagent attachment sites and thus the putative locations of the peptides that were involved in the analyzed interactions. 

For the interaction with VTR, four peptides on *C. albicans* enolase and three peptides on *C. tropicalis* enolase were identified, suggesting that the sequence fragment _334_IKTAIEK/IKKAIEK_340_, and _236_KAGYKGKVGIAMDVASSEFYKDGKYDLDFK/ QAGHTGKVKIAMDPASSEFFKDGKYDLDFK_265_ contribute to the VTR-contact area in both enolases. For the enolase-FN pairs, four peptides from *C. albicans* enolase and two from *C. tropicalis* enolase were identified, indicating a common sequence _245_IAMDVASSEFYKDGK/IAMDPASSEFFKDGK_259_ in the FN-binding region in both enzymes. For the interaction of HPG, four and three peptides on *C. albicans* and *C. tropicalis* were found, with the common sequences _107_LGANAILGVSLAAA_120_, _237_AGYKGK/AGHTGK_242_ and _245_IAMDVASSEFYKDGK/IAMDPASSEFFKDGK_259_ putatively identified as contributing to the HPG-binding area. We finally concluded that the long sequence _236_KAGYKGKVGIAMDVASSEFYKDGK/QAGHTGKVKIAMDPASSEFFKDGK_259_ is a consensus site for enolase binding of all three human proteins studied. 

An inverted setup for cross-linking experiments, i.e., one in which first enolase reacted with sulfo-SDAD in the dark, followed by linking to human protein under UV light, was applied for VTR and HPG binding by candidal enolases, revealing one peptide from VTR—_198_DVWGIEGPIDAAFTR_212_—and two from HPG—_48_ECAAKCEE_55_ and _520_TNPRAGLE_527_—that putatively interact with candidal enolases. 

### 2.6. Molecular Modeling of the C. albicans Enolase Interactions with VTR, HPG and Fragments of FN

Enolase fragments involved in the interactions with human proteins—VTR, FN and HGP—were mapped and structural models for these interactions were developed using the ClusPro 2.0: protein–protein docking software (Boston University). The docking was performed for the *C. albicans* enolase dimeric structure (obtained using homology modelling based on the template PDB ID: 1EBH [58]) and the full-length HPG structure (Research Collaboratory for Structural Bioinformatics Protein Data Bank, PDB ID: 4DUU [59]), FN fragment (PDB ID: 3M7P [60]) and the VTR structure obtained using the Schrödinger Prime software (LLC) based on the multiple templates (PDB ID: 3C7X, 1OC0, 2JQ8 and 3BT1 [61,62,63,64])

**Figure 5:**
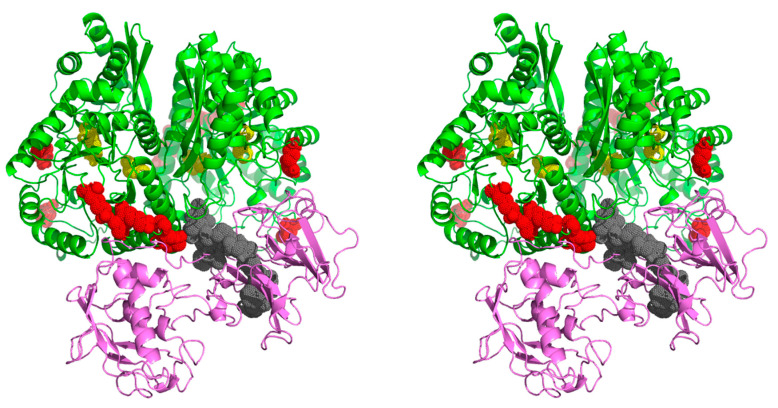
Proposed model of the interaction between *C. albicans* enolase (green) and human VTR (purple). The docking was performed for the *C. albicans* enolase dimeric structure (template PDB ID: 1EBH [58]) and VTR structure obtained based on multiple templates (PDB ID: 3C7X, 1OC0, 2JQ8 and 3BT1 [61,62,63,64]). The enolase peptides and the VTR peptide identified in the chemical mapping experiments are indicated in red and gray, respectively. The active site residues of the catalytic center of enolase are indicated in yellow. The 3D molecular model is presented in a wall-eyed stereo view. An additional file Enolase_VTR.mpg with the PyMOL-generated video presentation of this model is attached to the article together with the Supplementary Materials (Video S1).

The model presented in Figure 5 indicates that the four identified enolase peptides and one VTR peptide, denoted in red and grey, respectively, are indeed involved in the interactions between these proteins. Interestingly, the active center of enolase (marked in yellow) does not interact with VTR and is unoccupied in this model, consistent with the above results indicating that the binding to host proteins does not affect the enzymatic activity of enolase. 

**Figure 6:**
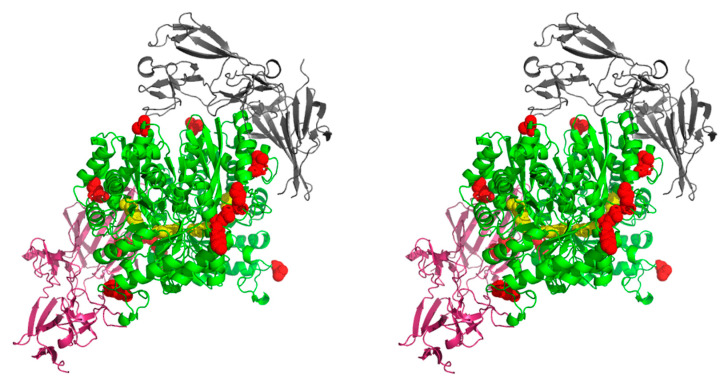
Stereo image (wall-eyed) of the interaction between *C. albicans* enolase and human FN. The docking was performed for the *C. albicans* enolase dimeric structure (template PDB ID: 1EBH [58]) and FN fragment (PDB ID: 3M7P [60]). The two models for the protein-protein interaction between a human FN fragment (black and pink) and *C. albicans* enolase (green with active site residues of the catalytic center indicated in yellow) were generated using ClusPro 2.0: protein-protein docking software. The enolase peptides identified in the chemical mapping experiments are marked in red. Additional files Enolase_FN_1.mpg and Enolase_FN_2.mpg with the PyMOL-generated video presentations of these models are attached to the article together with the Supplementary Materials (Videos S2 and S3).

In the case of enolase interactions with FN, chemical cross-linking experiments did not allow us to map the enolase-binding region due to the size, strong glycosylation and still unresolved structure of the whole FN protein. A 308 amino acid fragment of FN (PDB structure: 3M7P [60]) was used for molecular modeling, and two different models were thereby proposed for its interaction with enolase. The models were verified by the direct interaction of the FN fragment with the peptides identified on enolase (Figure 6).

**Figure 7:**
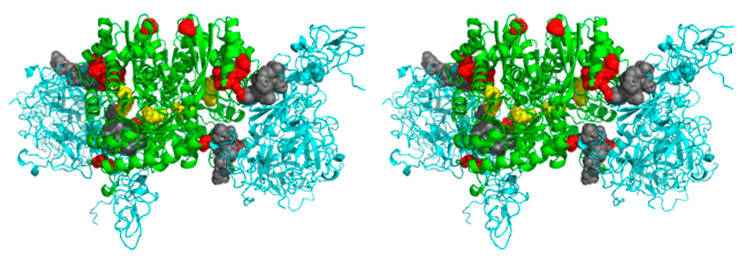
Proposed model of the *C. albicans* enolase interaction with human HPG. The docking was performed for the *C. albicans* enolase dimeric structure (template PDB ID: 1EBH [58]) and HPG structure (PDB ID: 4DUU [59]). The enolase dimer is indicated in green and HPG is denoted in blue. The enolase and HPG peptides identified in the chemical mapping experiments are marked in red and gray, respectively. The active site residues of the catalytic center of the enolase are highlighted in yellow. The 3D molecular model is presented in a wall-eyed stereo view. An additional file Enolase_HPG.mpg with the PyMOL-generated video presentation of this model is attached to the article together with the Supplementary Materials (Video S4).

**Figure 8:**
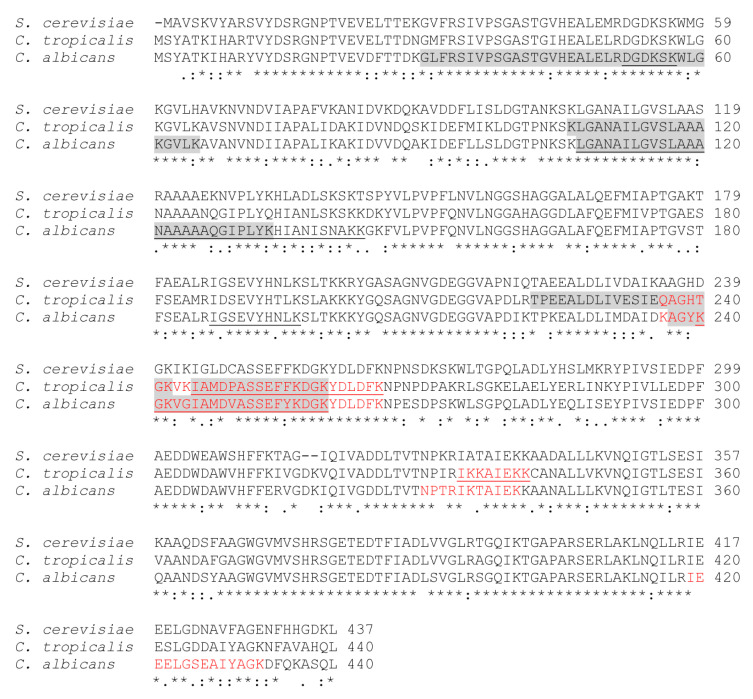
Comparison of the putative human protein binding sites on the *C. albicans*, *C. tropicalis*, and non-pathogenic *S. cerevisiae* enolases. Alignment of the enolase amino acid sequences from *C. albicans* (accession number P30575), *C. tropicalis* (accession number C5MD83) and *S. cerevisiae* (accession number P00929). Peptides identified during chemical crosslinking involved in the interaction with HPG are marked in a gray background, with VTR are marked with red letters, and with FN are highlighted by underlines.

As mentioned above, five peptides on *C. albicans* enolase were identified from cross-linking analysis of the interaction with HPG (marked in red in Figure 7), but the subsequent molecular modeling did not confirm any involvement in this interaction for one of them—_30_GLFRSIVPSGASTGVHEALELRDGDK_55_. Interestingly, a comparison of the amino acid sequences of fungal enolases indicated that this sequence region is identical in the *C. albicans* and *S. cerevisiae* enzymes (Figure 8). 

### 2.7. The Sequence Fragment _235_DKAGYKGKVGIAMDVASSEFY_255_ of C. albicans Enolase is a Major Determinant of Its Interactions with Human Proteins

The full amino acid sequence of the enolase from *C. albicans* shows a high degree of homology to the *C. tropicalis* (82.5%) and *S. cerevisiae* (77.5%) enolases (Figure 8). Considering the results of our chemical cross-linking mapping of enolase interactions with VTR, FN and HPG, combined with the sequence alignments shown in Figure 8, a single enolase peptide fragment was found to be involved in the interaction with all three human proteins tested in our current study. Interestingly, this fragment—_235_DKAGYKGKVGIAMDVASSEFY_255_—is significantly different from the corresponding sequence in *S. cerevisiae* enolase which, as mentioned above, interacts weaklier with human proteins. 

**Figure 9:**
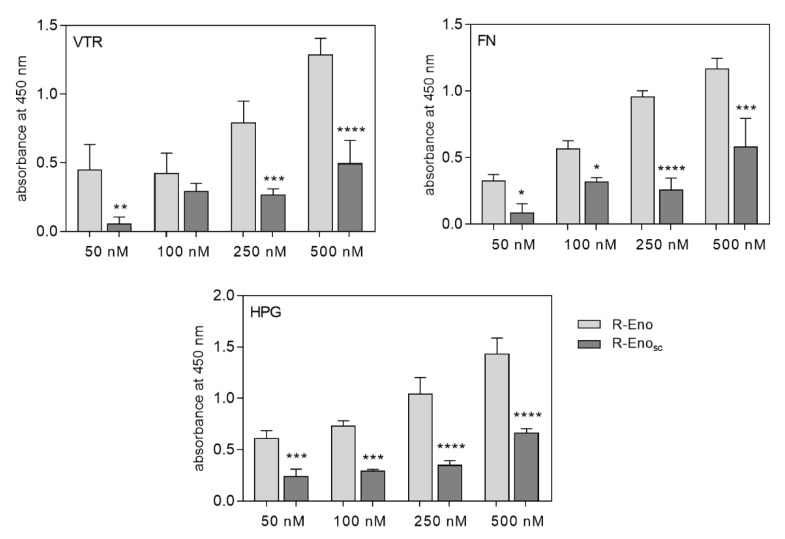
Binding of biotin-labeled recombinant fungal enolase to microplate-immobilized VTR, FN and HPG. Microplate wells coated with 3 pmoles of VTR, FN or HPG were filled with 50 µL of biotin-labeled recombinant enolases in increasing concentrations. The absorbance intensities were measured after the washing of unbound proteins. The results obtained during the control experiment (non-human protein surface blocked with 3% BSA solution) were subtracted from the total binding measurements. The bars represent the mean values ± standard deviation (3 determinations). Statistical significance was determined using one-way ANOVA; * *p* < 0.05, ** *p* < 0.01, *** *p* < 0.001, **** *p* < 0.0001.

To evaluate the significance of this fragment, recombinant proteins were obtained i.e., full-length *C. albicans* enolase (R-Eno) and *C. albicans* enolase in which the _235_DKAGYKGKVGIAMDVASSEFY_255_ sequence was replaced with the corresponding sequence of *S. cerevisiae* enolase (R-Eno_sc_)—_234_KAAGHDGKIKIGLDCASSEFF_254_. After purification, the enolases were labelled with biotin and analyzed for their binding to microplate-immobilized human proteins. Based on these tests, a reduction in binding by more than 50% was found for R-Eno_sc_ compared to R-Eno, for which the obtained level of human protein binding was given a reference value of 100% (Figure 9). These experiments, independently of other data presented in this study, confirmed the important role of this sequence fragment in the interactions of candidal enolases with human host proteins.

## 3. Discussion

The numerous intracellular proteins, including housekeeping enzymes, chaperones, translation factors, DNA-binding proteins and many others, that are non-conventionally secreted and reside on the cell surface where they act as receptors for soluble proteins or small molecules, comprise the largest subset of moonlighting proteins [65]. Enolase is a prominent representative of this multifunctional protein class and has been reported to be exposed on the surface of diverse prokaryotic and eukaryotic cells. The cell surface-bound form of human enolase can contribute to various non-infectious pathological conditions [1,2,3]. The enolases expressed on bacterial [21] and eukaryotic [23,24] pathogens have been confirmed as virulence factors. 

The presence of enolase on the cell wall of Candida spp. has been reported in a number of previous studies [25,26,27,38,40,66,67,68]. However, the mechanisms underlying the presence and relatively stable attachment of candidal enolase at the cell wall have not yet been identified. Current hypotheses include a sorting role of the 169 amino acid N-terminal domain of this enzyme [25] or the transport of enolase among the cargo of extracellular vesicles [69].

We aimed in our current study to structurally characterize the interactions between candidal enolases and selected human host proteins that would reflect two of the major functions that enolase and numerous other moonlighting proteins perform on the cell surfaces of various microbial pathogens, i.e., the adhesion to human host tissues and the interference with host homeostatic/hemostatic proteolytic cascades (reviewed in: [7]). The generally accepted model proteins used to probe for moonlighting adhesion functions have been proteinaceous ECM components such as FN, VTR, collagen, LAM (for examples see [70,71,72]). VTR and FN are also present in blood. Although only in qualitative terms, the binding of these host ligands by candidal enolase has been described by us previously [45]. On the other hand, the binding of HPG by cell-surface proteins is a very general phenomenon, observed in a variety of mammalian cells, and in eukaryotic and bacterial pathogens (for a recent review see [3]). Our present study supports the previously suggested hypothesis that candidal enolase contributes to the activation of fibrinolysis and plasmin-dependent ECM degradation during candidiasis [46,47,48]. We had further noted in this regard that enolase had been confirmed as one of multiple HPG-biding proteins at the candidal surface [48,50,51].

We used several preparations of enolase in our current experiments: natural enolases purified from the cytosol or cell wall of *C. albicans* and *C. tropicalis*, and, for comparative purposes, a commercially available natural enolase from *S. cerevisiae*. Initially, using tests with whole candidal cells, we confirmed a significant (20–40%) contribution of cell surface enolase to the total binding of VTR, FN or HPG (Figure 1), despite the known co-occurrence of a vast array of both true (i.e., GPI-anchored) and moonlighting adhesins at the candidal cell wall. At the pure protein-protein level, the binding of isolated candidal enolases to VTR, FN and HPG was analyzed using two different methods. For comparative purposes, a semi-quantitative estimation of the overall binding level using a microplate test for the interaction of biotin-labelled enolase with polystyrene-immobilized human protein has usually been sufficient. In more rigorous evaluations, quantitative binding parameters (the association and dissociation rate constants *k_a_* and *k_d_*, and the equilibrium dissociation constant *K_D_*) were calculated from SPR measurements, thereby indicating relatively strong interactions between the fungal and human proteins, with the *K_D_* constants ranging from 10^−7^ to 10^−8^ M. The applied method of chip surface regeneration (1 M NaCl) suggests that electrostatic forces can be the major determinants of the protein-protein interactions studied. The dissociation constants were within the same range, as determined using the SPR method for the binding of HPG and FN to *Streptococcus suis* enolase [73], by biolayer interferometry for the interactions of *Aspergillus fumigatus* enolase with HPG [74], and by SPR measurements for the interactions of candidal enolases with the components of plasma kinin-forming system [68,75]. Somewhat surprisingly, as determined by the SPR method, the interactions of human proteins—HPG, FN, LAM, and kininogen—with dedicated adhesins covalently linked to the candidal cell wall—Als1, Als7, Hyr, Epa1, and Epa6—were not previously found to be stronger than the moonlighting enolase-dependent interactions [49,67,76,77,78]. This further supported the hypothesis that fungal cell wall-associated enolase plays a remarkable role in the adherence of fungal pathogens to human host tissues. 

One of the important findings in this part of our study was that the strengths of the interactions between all three human proteins we tested with cell surface-bound and cytosolic forms of candidal enolase were indistinguishable, thus excluding the possibility that the moonlighting adhesin function of enolase is acquired upon the transfer of this enzyme from the cytosol to the cell surface and possibly due to some significant changes to this protein’s covalent structure or conformation, and/or posttranslational modifications that occur exclusively for cell wall enolase. The candidal cell surface enolase has an identical molecular mass and specific enzymatic activity as the cytosolic form, thus confirming that all of the structural elements essential for the evolutionally conserved catalytic function are preserved following non-conventional enzyme secretion. An accidental glycosylation that could occur if enolase “illegally” enters the classical secretion membrane system [23] was also excluded. Several posttranslational modifications at specific amino acid residues were previously identified for human enolase in various severe diseases [79,80], and also in the enolase from the *Plasmodium falciparum* parasite [81]. Analogous modifications at corresponding residues in candidal enolases, even if they occur [40], are unlikely to underly the moonlighting adhesin function of the altered enzyme, as the recombinant *C. albicans* enolase produced in *E. coli* was shown to bind the three human proteins we analyzed in a comparable manner to the naturally occurring enzyme. Thus, we conclude that the structural determinants critical for the binding of host proteins by the enolase at the candidal cell surface are inherent also to the enolase molecules present in the cytosol.

We did not observe any remarkable differences in the VTR-, FN- and HPG-binding strength and capacity between the enolases from *C. albicans* and *C. tropicalis*. The moonlighting binding ability of candidal cell surface proteins towards various ECM/blood proteins was reported in our previous study in *C. parapsilosis* and *C. glabrata* [45,49,78]. Thus, we hypothesized in our present report that the moonlighting adhesion function of enolase would be conserved within the Candida genus. The amino acid sequences of *C. albicans* and *C. tropicalis* enolases are 82.5% identical. In some protein families, very small sequence differences can generate a moonlighting function of one member, in the absence of this property in the others [53,54]. It was evident from our present data that the differences between the amino acid sequences of the candidal enolases (Figure 8) are not sufficiently concentrated at the areas on this protein molecule that come into a tight contact with human VTR, FN or HPG in the formed complexes. 

On the other hand, the enolase of a non-pathogenic yeast that shares 77.5% identical amino acid residues in the sequence with *C. albicans* enolase, presented much weaker, if any, binding to VTR, FN and HPG. These observations support the hypothesis that the capability of enolase to interaction with host proteins is particularly strongly evolved in pathogenic yeasts and makes important contributions to their virulence. The sequence alignment of the enolases of Candida spp. and *S. cerevisiae* (Figure 8) did not initially reveal a candidate sequence fragment that can lie in the contact area between the enolase and human proteins, based on the absence of at least several specific amino acid residues in the non-pathogenic yeast enolase. 

The main aim of our current study was to present structural models for the interaction of candidal enolase with three human proteins that we chose to probe the major moonlighting functions of this intracellular/secreted enzyme at the pathogen cell surface. For this purpose, we used a three-step strategy involving: (i) mapping the peptide sequences, potentially involved in enolase interactions with human proteins by chemical cross-linking and mass spectrometric analysis; (ii) molecular modelling of VTR-, FN- and HPG-enolase complexes; and (iii) confirming the model by substituting, in the recombinant candidal enolase, the sequence critical for the interactions with human proteins with the corresponding peptide in *S. cerevisiae* enolase.

For assessing the interactions of enolase with human ECM proteins, no structural data have been yet published for this enzyme from any organism. To our knowledge, no such analyses regarding the binding of ECM components have been performed for any moonlighting protein. By chemical cross-linking analyzes, we identified a number of peptides that putatively interact with VTR and FN (Table 2), all containing positively charged lysine residues and located at some distance from the catalytic active site of enolase. Some of these seemed to contribute to the binding of VTR and FN. The longest sequence fragment that putatively interacted with both of these proteins was _236_KAGYKGKVGIAMDVASSEFYKDGK_259_. From the perspective of enolase as a ligand, for complex formation with VTR, our cross-linking data were sufficient to identify a VTR fragment that putatively comes into contact with enolase—_198_DVWGIEGPIDAAFTR_212_. 

In the next step of our analyses, using available bioinformatics platforms and taking into account the results of our earlier cross-linking-based mapping, structural models for the complexes of enolase with our chosen human host proteins were developed. The entire VTR molecule and an FN fragment (aa 297–604) were docked to candidal enolase dimeric molecules, modelled on the basis of the known crystal structure of enolase from *S. cerevisiae*, with two identical subunits facing each other in an anti-parallel manner [82]. In the finally developed models (Figure 5 and Figure 6), the contact region between enolase and the ECM proteins was clearly spaced from the enzyme active center. In the enolase-VTR complex, some peptides identified in the two proteins by cross-linking were found to make contact with each other, thus elegantly validating the model. In the case of VTR, the cross-linking experiments identified that the fragment _198_DVWGIEGPIDAAFTR_212_ that interact with fungal enolase are located in the central hemopexin-like domain (aa 131–342). This domain is known to bind numerous ligands such as heparin, type I collagen, β-endorphin, cholesterol sulphate, or complement complex. In addition, hemopexin-like repeats are known binding sites for *Streptococcus pyogenes* cells [83]. In our present models, as could be expected, the spots on enolase that face VTR and FN extensively overlap. 

Being a universal binding phenomenon that contributes to the virulence of many pathogenic microbes [84,85,86,87], the interactions of enolase with HPG has been much better characterized in molecular terms. Two C-terminal lysine residues were suggested to be the main sites involved in HPG binding by the enolases of humans and various pathogens [88]. Confirmations of their role were obtained by site-directed mutagenesis of the enolases of several bacterial species where the substitutions of leucine or alanine residues for the critical lysine residues resulted in a two-fold decrease in HPG binding [87,88,89,90]. In addition, the role of an internal HPG-binding motif in enolases—_248_FYDKERKVY_256_—was proposed for many organisms, including parasites and bacteria, however, in most cases, only on the basis of amino acid sequence comparisons [91,92,93,94,95]. For two species only—*Streptococcus pneumoniae* and *Leishmania mexicana*, the role of this internal enolase motif was experimentally supported by a competitive inhibition assay with synthetic peptides and also by site-directed mutagenesis of the binding motif [91,92]. The replacement of three amino acid residues in the selected fragment of the bacterial enolase (Lys_251_Leu, Glu_252_Gly and Lys_254_Leu substitutions) reduced HPG binding to 44% of the wild-type level, whilst the additional deletion of the C-terminal lysine residues did not further reduce this binding. Hence, this internal motif within bacterial enolases appears to be crucial in the binding process [91]. The fact that the same HPG binding motif has been identified in different species suggests that it may also be relevant for other pathogenic organisms [85].

In our current cross-linking analyses, we identified four candidal enolase peptides that seemed to interact with HPG, all containing lysine and, occasionally, arginine residues (Table 2). Similar to the enolase sites that were responsible for complex formation with VTR and FN, these peptides were located outside of the enzymatic active center. Interestingly, the _237_AGYKGKVGIAM DVASSEFYKDGK_259_ sequence was again identified as a common long fragment essential for enolase binding to all of the human proteins under study. 

For molecular modeling of the candidal enolase-HPG complex, the full length HPG protein was docked to the modelled dimeric candidal enolase, and the results of cross-linking experiments were also considered. The model obtained from this (Figure 7) shows that HPG adheres to enolase at a similar area as VTR and FN, far away from the enzyme active center. It was not surprising that there would be little room for evolutional variations to accommodate different and large proteinaceous ligand binding at very different areas of the enolase molecule if the evolutionally conserved catalytic role of enolase in central metabolism was to be preserved. 

The binding of HPG to mammalian and bacterial cells was reported to be mediated by kringle domains that have an affinity for lysine [96,97]. One of the HPG peptides we here identified —_520_TNPRAGLE_527_—is located within kringle domain 5, additionally confirming that the binding of HPG to *C. albicans* enolase is lysine dependent [92,98].

As mentioned above, *S. cerevisiae* enolase binds VTR, FN and HPG with a much lower affinity than the candidal enolases. Notably however, sequence comparisons for these proteins (Figure 8) revealed a similarity between the major identified sequence fragments, with differences in only few amino acids. At the positions of the lysine residues in *C. albicans* enolase important for HPG binding (Lys_236_) and VTR/FN binding (Lys_240_), a negatively charged aspartic acid residue and non-polar alanine residue is present in the *S. cerevisiae* enzyme. Finally, to confirm that role of the enolase sequence fragment—_235_DKAGYKGKVGIAMDVASSEFY_255_—in the binding of VTR, FN and HPG, this sequence was substituted in a recombinant candidal protein for the corresponding *S. cerevisiae* enolase sequence. At least a two-fold reduction of VTR-, FN- and HPG-binding was observed in the resulting mutant protein compared with the wild-type protein (Figure 9). A complete abolishment of the interactions studied was not something to be expected, as the high affinity with which candidal enolase binds human proteins seems to be an additive effect i.e., involving the participation of all binding regions with a particular role of positively charged lysine residues. A confirmation of this hypothesis may be the Thr_142_ to Lys_143_ exchange between two recombinant enolases resulting in the location of two lysine residues side-by side in the candidal enzyme. Thus, our current study has provided molecular data that may fit into a previously proposed general rule for the interactions of moonlighting proteins with their non-canonical ligands [53] i.e., that relatively small changes in the enolase sequence may contribute to the engagement of this protein as a contributing factor in the pathogenic features of Candida spp. yeasts.

## 4. Materials and Methods 

### 4.1. Yeast Strains and Culture Conditions

The *C. albicans* strain ATCC^®^ 10231™, and *C. tropicalis* strain T1 (ATCC^®^ MYA-3404™) were purchased from American Type Culture Collection (Manassas, VA, USA). Yeast cultures were incubated in YPD medium containing 1% yeast extract, 2% soybean peptone and 2% glucose at 30 °C for 16 h with shaking at 170 rpm (MaxQ 4000, Thermo Fisher Scientific, Waltham, MA, USA). To induce the hyphal or pseudohyphal forms of *C. albicans* and *C. tropicalis*, respectively, further culturing was carried out in RPMI 1640 medium at 37 °C for 72 h with shaking at 170 rpm. 

### 4.2. Commercial Proteins

Human VTR was purchased from R&D Systems (Minneapolis, MN, USA), human FN, HPG and *S. cerevisiae* enolase from Sigma-Aldrich (St. Louis, MO, USA), bovine serum albumin (BSA) from BioShop Canada Inc. (Burlington, ON, Canada), β-1,6-glucanase from Takara Bio Inc. (Otsu, Shiga, Japan), trypsin and endoproteinase Glu-C from Promega (Madison, WI, USA), horseradish peroxidase-conjugated streptavidin (SA-HRP) solution from MP Biomedicals (Solon, OH, USA), monoclonal mouse anti-fungal enolase antibody from Invitrogen (Thermo Fisher Scientific) and enterokinase from MyBioSource (San Diego, CA, USA).

### 4.3. Purification of C. albicans Surface-Exposed Enolase 

After culturing *C. albicans* in RPMI 1640 medium, preparations of cell surface-exposed proteins were obtained by β-1,6-glucanase treatment according to our previously described method [49]. Briefly, the first step in the purification procedure was ion-exchange chromatography. The isolated fungal protein mixture (10 mL), pre-dialyzed against 20 mM Tris-HCl buffer, pH 8.0, was loaded onto a Resource^TM^ Q 1 mL column (GE Healthcare, Uppsala, Sweden) equilibrated with the same buffer. Adsorbed proteins were then eluted in a linear gradient of 0–0.5 M NaCl (20 mL) at a flow rate of 1 mL/min [49]. The collected fractions were analyzed by SDS-PAGE and those that showed strong bands corresponding to the molecular mass of enolase (47 kDa) were subjected to the next purification step—gel filtration on a TSK G 3000 SW (21.5 mm × 30 cm, particle size 13 µm) column (Tosoh Bioscience, King of Prussia, PA, USA). The column was eluted with 0.1 M sodium sulphate/0.1 M sodium dihydrogen phosphate, pH 6.7, and the collected fractions were characterized by SDS-PAGE, and analyzed by LC-MS/MS using our previously published protocols [49]. The enolase-containing fractions were finally subjected to chromatofocusing on a MonoP HR 5/20 column (GE Healthcare), equilibrated with 25 mM Bis-Tris-iminodiacetic acid buffer, pH 7.1, and further eluted in a linear gradient of 10% Polybuffer 74 in 20 mM Bis-Tris-iminodiacetic acid buffer pH 7.1 (Sigma-Aldrich), in a total volume of 20 mL, at a flow rate of 0.5 mL/min. The collected fractions were next individually loaded onto a Superdex 200 HR 10/50 column (Amersham Biosciences, Uppsala, Sweden) in 20 mM Tris-HCl pH 6.7 at a flow rate of 0.5 mL/min to separate the proteins from ampholytes. The enolase purity and identity were confirmed by SDS-PAGE and LC-MS/MS analysis. 

### 4.4. Purification of the Cytosolic Enolases from C. albicans and C. tropicalis

The fungal cytosolic enolases were purified using a previously described strategy [75], in accordance with the method of Ballantyne and Warmington, with some modifications [99] Briefly, Candida spp. cells were grown in YPD medium at 30 °C for 16 h with shaking at 170 rpm. After centrifugation (2-16KL, Sigma-Aldrich, 1600× *g*, 3 min) and washing of the cell pellet twice with phosphate-buffered saline (PBS), pH 7.4, the cells were suspended in water with protease inhibitors (Complete Tablets EDTA-free, EASYpack, Roche, Penzberg, Germany) and sonicated in a Sonic Ruptor 250 Ultrasonic Homogenizer (Omni International, Tulsa, OK, USA) for 6 min, with the amplitude set at 0.5. The resulting protein solution was subjected to ammonium sulphate fractionation, with the first saturation to 65% and discard of the pellet, and the second saturation of the resulting supernatant to 100%. After centrifugation (13,000× *g*, 20 min), the supernatant was discarded and the pellet was dissolved in 20 mM Bis-Tris buffer, pH 6.4, dialyzed overnight against the same buffer, and separated on a MonoQ 1 mL column (Pharmacia Biotech, Piscataway, NJ, USA) using a linear gradient of 0.5–1 M NaCl (80 mL). Fractions with enolase activity (see below) were collected and dialyzed overnight against 10 mM sodium acetate buffer, pH 4.8. The final enolase purification was performed on a MonoS 1 mL column (GE Healthcare) and a linear gradient of 0–1 M NaCl (75 mL). The enolase purity and identity were confirmed by SDS-PAGE and LC-MS/MS analysis.

### 4.5. Expression and Purification of Recombinant C. albicans Enolase (R-Eno)

After culturing in YPD medium, the yeast cells were dissolved in TRI^®^Reagent (Sigma Aldrich). After adding glass beads (425–600 µm, Sigma Aldrich), the cells were ruptured using the FastPrep device Precellys Evolution (Bertin Technology, Montigny-le-Bretonneu, France) for 2 cycles at 45 s each and a 6.0 RPM speed. The total RNA was extracted in accordance with the protocol provided with the TRI^®^Reagent, followed by the synthesis of the cDNA strand using 2 µg total RNA, 0.5 µg of oligonucleotide (dT)18 primer and 200 U of MLV reverse transcriptase (Moloney murine leukemia virus reverse transcriptase; Promega). The complete coding region of the *C. albicans* enolase gene was amplified by PCR using the C1000 Touch Thermal Cycler (Bio-Rad, Hercules, CA, USA), using the specific primers: forward, 5′GGTGATGATGATGACAAGATGTCTTACGCCACTAAAATC3′ and reverse, 5’GGAGATGGGAAGTCATTATTACAATTGAGAAGCCTTTTG3’. The reaction conditions were as follows: 95 °C for 2 min, followed by 30 cycles of 95 °C for 30 s, 52 °C for 30 s, 74 °C for 3 min and 74 °C for 10 min. PCR products were electrophoretically verified in a 1% agarose gel. 

Enolase expression constructs were prepared using the aLICator LIC Cloning and Expression Kit (Thermo Fisher Scientific) according to the manufacturer’s instructions. Briefly, PCR products were purified using the Gel-Out Concentrator Kit (A&A Biotechnology, Gdynia, Poland) and then incubated with T4 DNA Polymerase in LIC buffer for 5 min. The reaction was stopped by the addition of EDTA to a final concentration of 30 mM. The pLATE51 expression vector harboring an N-terminal 6xHis tag and an enterokinase protease cleavage site was next added and the mixture was incubated for 5 min at room temperature. This annealed mixture was used directly to transform *E. coli* TOP10 (Thermo Fisher Scientific) cells. Positive transformants were selected for their resistance to ampicillin (100 μg/mL). Plasmids were isolated using the Mini Plasmid kit (A&A Biotechnology), and after sequence verification (Genomed, Warsaw, Poland) were used to transform chemically competent Rosetta^TM^ 2 (DE3) *E. coli* cells. The overnight culture was inoculated into tryptic soy broth (TSB) medium containing ampicillin (100 µg/mL) and chloramphenicol (34 µg/mL). The resulting bacterial cultures were grown at 37 °C to the middle logarithmic phase and the expression of *C. albicans* enolase was induced with 1 mM isopropyl-β-D-thiogalactopyranoside (IPTG) whilst maintaining the cells at 30 °C for 3 h. After cell lysis by sonication (10 min, 0.8 amplitude), the supernatant and pellet fractions were separated by centrifugation (20,000× *g* at 4 °C for 30 min). The recombinant protein was purified using a strategy similar to that previously described [68]. Briefly, during the first step of purification, the supernatant, containing the recombinant protein (His6-enolase) was loaded onto an Ni–NTA Sepharose High Performance affinity matrix 2 mL column (GE Healthcare). Fractions containing enolase were diluted to a final protein concentration of 0.5 mg/mL with an appropriate volume of enteropeptidase cleavage buffer. In accordance with the manufacturer’s protocol, the enzyme was added in a ratio of 50 µg protein:1 U enterokinase. After 20 h, the mixture was again loaded onto an Ni-NTA Sepharose column and the tag-free enolase was subjected to ion-exchange chromatography on a MonoQ 1 mL column (Pharmacia Biotech). Finally, the purity of the selected fractions was verified by SDS-PAGE and an activity assay was performed (see below). 

### 4.6. DNA Construct for R-Eno_sc_ Expression

The construct used for R-Eno_sc_ expression was prepared by substitution of a fragment of *C. albicans* enolase coding sequence with a corresponding coding sequence fragment of *S. cerevisiae* enolase. The final recombinant coding sequence of *C. albicans* enolase with the coding sequence fragment of *S. cerevisiae* enolase (nucleotide sequence: AAGGCTGCTGGTCACGACGGTAAGATCAAGATCGGTTTGGACTGTGCTTCCTCTGAATTCTTC, amino acid sequence: _234_KAAGHDGKIKIGLDCASSEFF_254_), together with a fragment encoding the WELQut protease cleavage site upstream of the enolase coding sequence, was synthesized and cloned into the existing open reading frame of the pETDuet-1 vector between the BamHI and XhoI restriction sites (GenScript, Piscataway Township, NJ, USA). The obtained plasmid construct was used for bacterial transformation and positive transformants were selected. The plasmid was isolated using a Plasmid Mini Kit (A&A Biotechnology) and used to transform chemically competent RosettaTM 2 (DE3) cells (Novagen). The further steps taken in relation to protein expression and purification were analogous to those used for *C. albicans* recombinant enolase (R-Eno).

### 4.7. Enolase Activity 

Enolase activity was analyzed using a colorimetric assay kit (Sigma-Aldrich) following the manufacturer’s instructions. In the assay, enolase converts D-2-phosphoglycerate into phosphoenolpyruvate, which further reacts with peroxidase substrate to form a colored product with an absorbance at 570 nm, which is measured for 1 h with 3 min intervals (using a Synergy H1 microplate reader; BioTek, Winooski, VT, USA). In one experiment, a modification was introduced i.e., before measuring its activity, enolase at a final concentration of 20 nM was incubated with VTR, FN, and HPG at a 1:1 molar ratio for 60 min in PBS at 37 °C, with gentle shaking. Wells without human protein constituted the reference control equating to 100% enolase activity. One unit (U) of enolase is the amount of enzyme that generates 1.0 µmole of H_2_O_2_ per minute at pH 7.2, 25 °C.

### 4.8. Labeling of Human Proteins and Purified Enolases

For biotin labeling of FN (FN-Bt), VTR (VTR-Bt), HPG (HPG-Bt), R-Eno (R-Eno-Bt) and R-Eno_sc_ (R-Eno_sc_-Bt), a solution of *N*-hydroxysuccinimide ester of biotin (NHS-biotin, Sigma-Aldrich) in dimethylformamide (1 mg/100 µL) was added, maintaining a ratio of 10 µg NHS-biotin per 50 μg protein. The prepared reaction mixture was incubated for 4 h at 4 °C and then dialyzed against PBS at 4 °C for 24 h. 

### 4.9. Competition of Human VTR, FN and HPG with Soluble Enolase or Anti-Fungal Enolase Antibody for the Binding to C. albicans or C. tropicalis Cells

Competition binding assays were performed in MaxiSorp 96-well microtiter plates (Nunc, Roskilde, Denmark), in which Candida spp. cells (1 × 10^6^ cells per well) were grown in RPMI 1640 medium for 3 h at 37 °C. After each step, the cells were washed three times with 200 µL PBS, pH 7.4, additionally containing 1% BSA. The unoccupied well surface was blocked with 300 µL of 3% BSA in PBS for 1 h at 37 °C. Subsequently, 2 pmoles of VTR-Bt, FN-Bt or HPG-Bt (40 µL) and 24 pmoles of unlabeled purified enolases (10 µL) were added to the wells together, and the plate was incubated for 1.5 h at 37 °C with gentle mixing. The amount of bound biotinylated ECM protein was determined using SA-HRP and the peroxidase substrate 3,3′5,5′-tetramethylbenzidine (TMB) (Sigma-Aldrich), as described previously [100]. The 100% relative binding control reference constituted the wells in which enolase samples (10 µL) were replaced with PBS (10 µL).

The competition assay with antibodies was performed similarly with some minor modifications. Briefly, after blocking the unoccupied wells with 3% BSA, 1 µg/µL anti-fungal enolase antibody solution was added to the fungal cells. The mixture was then incubated for 1 h at 37 °C. After washing three times with PBS, solutions of biotinylated human proteins were added (250 nM in a total volume of 50 µL per well) and incubated for 1.5 h at 37 °C. 

### 4.10. Semi-Quantitative Analysis of Enolase Binding to Microplate-Immobilized VTR, FN, and HPG 

The binding assays for *C. albicans*, *C. tropicalis* and *S. cerevisiae* enolases, and the recombinant enolases—R-Eno and R-Eno_sc_, were also performed using MaxiSorp 96-well microtiter plates (Nunc) in which the human proteins were immobilized via an overnight incubation at 4 °C (3 pmol protein per well). Following this and all subsequent steps, the wells were washed three times with 1% BSA in PBS. The unoccupied surface in each well was blocked overnight with 3% BSA in PBS at 4 °C. Samples of biotinylated enolase solutions at increasing concentrations in the range of 10–500 nM (50 µL in PBS) were then added to the wells and incubated for 1.5 h at 37 °C. After washing out the unbound proteins, the binding levels were detected with the SA-HRP/TMB system. 

### 4.11. SPR Characterization of Fungal Enolase Binding to VTR, FN and HPG

Kinetic and thermodynamic analyses of the binding of fungal enolases to VTR, FN and HPG were carried out with a BIACORE 3000 system (GE Healthcare). First, for the activation of carboxyl groups present on the CM5 sensor chip (GE Healthcare), an Amine Coupling Kit (GE Healthcare) was used, i.e., a mixture of 50 mM 1-ethyl-3-(3-dimethylaminopropyl)carbodiimide (EDC) and 200 mM *N*-hydroxysuccinimide (NHS). Next, the immobilization of surface or cytosolic *C. albicans* enolases and cytosolic *C. tropicalis* enolase was performed at 25 °C with a flow rate of 10 µL/min in an acetate buffer at pH 4.5 to a level of 300 RU units. In the final step, carboxyl groups not involved in protein immobilization were blocked with 1 M ethanolamine solution, pH 8.5. The binding levels/ strength analyses for VTR, FN or HPG were performed at 25 °C in a buffer containing 10 mM HEPES, 150 mM NaCl and 0.005% (*w*/*v*) P20 surfactant, pH 7.4. After introducing the proteins above the chip surface, a 2-min association and 2-min dissociation occurred at a flow rate of 30 µL/min. Between the binding cycles, the chip surface was regenerated by the injection of 1 M NaCl for 30 s at a flow of 30 µL/min. BIAevaluation 4.1.1 software (GE Healthcare) was used to determine binding parameters obtained based on a global fit with a simple Langmuir model (1:1), with a baseline drift and Rmax locally.

### 4.12. Mapping of the Fungal Enolase Fragments Involved in the Interactions with VTR, FN and HPG by Chemical Cross-Linking 

#### 4.12.1. Chemical Cross-Linking

VTR, FN or HPG (20 µg in 100 µL PBS, pH 7.4) proteins were incubated in the dark at 4 °C for 2 h with 0.5 mM sulfo-SDAD (Thermo Fisher Scientific). After stopping the reaction with 50 mM Tris for 15 min, the excess reagent was removed by dialysis against PBS at 4 °C overnight in the dark. The tested human proteins were incubated with enolase (20 µg in 100 µL PBS, pH 7.4) at 37 °C for 1 h in the dark with gentle shaking. The samples were then placed on ice and exposed to UV radiation (365 nm) for 15 min (6W, Vilber Lourmat). Covalently linked pairs of human protein–enolase were dialyzed overnight against 25 mM ammonium bicarbonate buffer (NH_4_HCO_3_) at 4 °C. The protein complexes were then reduced with 50 mM dithiothreitol at 60 °C for 60 min and alkylated with 55 mM iodoacetamide at room temperature for 45 min in the dark. Next, 2 µL of trypsin solution (10 ng/µL in 25 mM NH_4_HCO_3_, pH 8.0) were added for 3 h, followed by additional 2 µL of trypsin solution for an overnight incubation at 37 °C. After stopping the reaction with 2 M HCl for 5 min, samples were centrifuged (15 min, 12,000 rpm), dried in a Speed-Vac (Martin Christ, Osterode am Harz, Germany) and frozen at −20 °C until further use. Occasionally, endoproteinase Glu-C was used for the digestion instead of trypsin. Analogous experiments were carried out in a reverse order, where first enolase was linked with sulfo-SDAD and then allowed to interact with human VTR or HPG proteins. 

#### 4.12.2. Mass Spectrometric Analysis

After resuspending in 10% acetonitrile with 0.1% formic acid, the peptides were analyzed with a HCTUltra ETDII ion-trap mass spectrometer equipped with an electrospray ionization ion source (Bruker, Bremen, Germany) and coupled to an ultra-high-performance liquid chromatograph Dionex Ultimate 3000 system (Thermo Fisher Scientific). All peptides were separated on a 100 mm  ×  2.1 mm Aeris 3.6 µm PEPTIDE XB C18 column (Phenomenex, Torrance, CA, USA), with a gradient of 10–60% of 0.1% formic acid in 80% acetonitrile, at a flow rate of 0.2 µL/min. Mascot Generic format (.mgf) files were generated by pre-processing the raw data with Data Analysis 4.0 software (Bruker). The obtained results were analyzed using the Mass Matrix PC server Version 4.3, as previously described [101]. The data set was then searched using Mass Matrix against the Swiss Prot human databases and NCBI or Swiss Prot fungal database with the following searching parameters: enzyme, —trypsin or endoproteinase Glu-C; missed cleavage, 3; peptide length, 4 to 50 amino acid residues; mass tolerances, −0.6 Da; fragmentation, —CID (collision-induced dissociation); and score thresholds of 4 for the pp (statistical scores evaluating a peptide match based on the number of matched product ions in the experimental spectrum), pp2 (statistical scores based on the total abundance of matched product ions in the experimental spectrum) and 1.3 for pptag (a statistical score that evaluates the match based on its color tags) scores. The chemical formula of the sulfo-SDAD between protein and sulfur is specified as C_7_OSNH_10_ with a monoisotopic mass of 156.048 Da in Mass Matrix. The windows version of the program is available at http://www.massmatrix.net/download/.

### 4.13. Competition between Human VTR and HPG for the Binding to C. albicans Enolase 

This competition assay was again performed in MaxiSorp 96-well microtiter plates (Nunc, Roskilde, Denmark) where VTR was immobilized by overnight incubation at 4 °C (3 pmol protein per well). The unoccupied surface in each well was blocked with 0.5% BSA in PBS at 37 °C for 3 h. After each step, the wells were washed three times with 1% BSA in PBS. Solutions of 40 nM biotinylated enolase (25 µL) and 400 nM HPG (25 µL) in PBS were added to the wells together and the plate was incubated for 1.5 h at 37 °C with gentle mixing. After washing out the unbound proteins, the amount of bound biotinylated enolase was determined using the SA-HRP/TMB detection system. The 100% relative binding of enolase reference was generated using wells in which the human protein (25 µL) was replaced with PBS (25 µL). In addition, a second variation of the experiment was also performed, in which magnesium ions (Mg^2+^) at a concentration of 5 mM were used at each stage.

### 4.14. Alignment of the Amino-Acid Sequences of C. albicans, C. tropicalis, and S. cerevisiae Enolases

Fungal enolase sequences were aligned and analyzed using Clustal Omega (1.2.4) [102]. *C. albicans* enolase (accession number P30575), *C. tropicalis* enolase (accession number C5MD83) and *S. cerevisiae* enolase (accession number P00929) were selected for these comparisons.

### 4.15. Model of the Interaction between C. albicans Enolase and HPG, VTR and FN

Homology modelling of C. *albicans* enolase (uniport ID: P30575) was carried out with the Modeller package v9.14 [103] using the crystal structure of *S. cerevisiae* enzyme (PDB ID: 1EBH [58]; the Research Collaboratory for Structural Bioinformatics Protein Data Bank, http://rcsb.org) as a template (Figure S2 in the Supplementary Material). The VTR structure (uniport ID: P04004, Vitronectin V65 subunit) was obtained by means of knowledge-based homology modelling method using multiple templates (PDB ID: 3C7X, 1OC0, 2JQ8 and 3BT1 [61,62,63,64]) in Prime software (Schrödinger Release 2019-1, Schrödinger LLC, New York, NY) (Figure S3 in the Supplementary Material). Lowest energy model with loop refinement was generated as the output and was used for subsequent analysis. Crystal structure of FN fragment (aa 297–604) and full-length HPG were taken from the PDB database (ID: 3M7P and 4DUU for FN and HPG, respectively [59,60]). Protein Preparation Wizard (Schrödinger, LLC) was used to prepare the final structure of proteins.

The model of interaction was elaborated using an on-line version of the protein-protein docking software ClusPro 2.0 (Boston University, https://cluspro.bu.edu). In the program, rigid structures were docked, and next from the group of 1000 structures with the lowest energy based on root mean-square deviation (RMSD) steric collisions were removed by energy minimization [104,105]. Finally, the resulting protein complexes were analyzed in the PyMOL Molecular Graphics System (Version 1.7.2.1; Schrödinger LLC) by comparing the distance between the experimentally selected amino acid residues of both proteins.

## 5. Conclusions

A certain amount of enolase—a cytosolic enzyme involved in evolutionally conserved metabolic pathways—appears at the cell surface of the pathogenic yeasts *C. albicans* and *C. tropicalis*, where it moonlights by contributing to pathogen adhesion to human host tissues, and to the activation of fibrinolysis and ECM degradation. In our present study, the positive interactions of enolase with the human proteins VTR, FN and HPG, that underlie these pathogenicity-related moonlighting functions of candidal enolase, were characterized in structural terms.

The quantitative binding parameters determined by SPR method for the interactions of human host proteins with candidal enolase classified these interactions as relatively tight. The binding strengths were found to be comparable between human ligands, between cytosolic and cell surface-exposed *C. albicans* enolases, and between enolases from two Candida species. In contrast, the binding of VTR, FN and HPG by the *S. cerevisiae* enzyme was much weaker. 

The chemical cross-linking method was used to map the sites on enolase molecules that come into direct contact with human proteins. A long sequence _235_DKAGYKGKVGIAMDVASSEFYKDGK_259_ present in *C. albicans* enolase was suggested to contribute to the binding of all three human proteins studied. Models for these interactions were developed and indicated the sites on the enolase molecule for binding of human proteins. These sites were extensively overlapping for these ligands and well-separated from the catalytic activity center of enolase.

Our present analyses suggest that the cell surface-exposed forms of *C. albicans* and *C. tropicalis* enolases may play an important role in the adhesion to host proteins, whilst the cytosolic forms retain their basic intracellular enzymatic function in central metabolism. This dual role may be a form of adaptation of previously unused molecular fragments as new binding sites. Thus, internal enolase motifs identified as points of pathogenicity-related interactions with host proteinaceous ligands may be promising targets for future drug design. 

## Figures and Tables

**Table 1:** Kinetic and thermodynamic parameters for the interactions of *C. albicans* and *C. tropicalis* enolases with VTR, FN or HPG, determined by SPR measurements. The SPR-determined *k_a_*, *k_d_* and *K_D_* values were obtained for enolase as a ligand and for human proteins as an analyte. All parameters are presented with the standard error.

Human Protein	Enolase Origin	*K_D_* [M]	*k_a_* [1/Ms]	*k_d_* [1/s]
VTR	*C. albicans*, cell wall	3.11 × 10^−^^8^ ± 4.58 × 10^−^^10^	1.34 × 10^5^ ± 2.52 × 10^3^	4.17 × 10^−3^ ± 7.63 × 10^−5^
VTR	*C. albicans*, cytosol	5.53 × 10^−^^8^ ± 2.43 × 10^−^^9^	8.91 × 10^4^ ± 5.06 × 10^3^	4.93 × 10^−3^ ± 9.12 × 10^−5^
VTR	*C. tropicalis*, cytosol	6.83 × 10^−^^7^ ± 4.39 × 10^−8^	1.34 × 10^4^ ± 1.11 × 10^3^	9.15 × 10^−3^ ± 5.96 × 10^−4^
FN	*C. albicans*, cell wall	3.68 × 10^−^^8^ ± 3.26 × 10^−9^	6.03 × 10^4^ ± 1.45 × 10^3^	2.22 × 10^−3^ ± 6.74 × 10^−5^
FN	*C. albicans*, cytosol	4.28 × 10^−^^8^ ± 3.74 × 10^−9^	6.47 × 10^5^ ± 9.84 × 10^3^	2.77 × 10^−2^ ± 3.58 × 10^−3^
FN	*C. tropicalis*, cytosol	5.18 × 10^−^^8^ ± 8.54 × 10^−^^10^	5.48 × 10^4^ ± 8.41 × 10^2^	2.84 × 10^−3^ ± 6.04 × 10^−5^
HPG	*C. albicans*, cell wall	1.20 × 10^−7^ ± 4.25 × 10^−6^	5.22 × 10^5^ ± 4.84 × 10^4^	6.26 × 10^−2^ ± 2.26 × 10^−3^
HPG	*C. albicans*, cytosol	2.57 × 10^−7^ ± 4.17 × 10^−6^	2.70 × 10^5^ ± 1.92 × 10^4^	6.93 × 10^−2^ ± 1.30 × 10^−3^
HPG	*C. tropicalis*, cytosol	2.53 × 10^−7^ ± 2.06 × 10^−6^	5.77 × 10^4^ ± 6.84 × 10^3^	1.46 × 10^−2^ ± 2.18 × 10^−1^

VTR: vitronectin; FN: fibronectin; HPG: human plasminogen.

**Table 2:** Mass spectrometry identification of *C. albicans* and *C. tropicalis* enolase peptides involved in the interaction with VTR, FN and HPG.

Peptides of *C. albicans* Enolase	Peptides of *C. tropicalis* Enolase
VTR-Bnding	
_236_KAGYKGKVGIAMD_248_ _243_VGIAMDVASSEFYKDGKYDLDFK_265_ _330_NPTRIKTAIEK_340_ _419_IEEELGSEAIYAGK_432_	_236_QAGHTGKVKIAMDPASSE_253_ _245_IAMDPASSEFFKDGKYDLDFK_265_ _334_IKKAIEKK_341_
FN-Binding	
_52_DGDKSK_57_ _107_LGANAILGVSLAAANAAAAAQGIPLYKHIANISNAKK_143_ _187_IGSEVYHNLK_196_ _240_KGKVGIAMDVASSEFYKDGK_259_	_245_IAMDPASSEFFKDGKYDLDFK_265_ _334_IKKAIEKK_341_
HPG-Binding	
_30_GLFRSIVPSGASTGVHEALELRDGDK_55_ _56_SKWLGKGVLK_65_ _107_LGANAILGVSLAAANAAAAAQGIPLYK_133_ _237_AGYKGKVGIAMDVASSEFYKDGK_259_	_106_KLGANAILGVSLAAA_120_ _222_TPEEALDLIVESIEQAGHTGK_242_ _245_IAMDPASSEFFKDGK_259_

A sulfo-SDAD cross-linking reagent with a disulfide bridge was added to each human protein preparation and the whole mixture was incubated with fungal enolases. After the formation of a covalent bond for the fungal protein-human protein pairs, the disulfide bond was reduced and the released proteins were digested with trypsin or, occasionally, endoproteinase Glu-C. The obtained peptides were then analyzed using a Dionex Ultimate 3000 UHPLC system coupled to an HCTUltra ETDII mass spectrometer. Within the panel of selected peptides, amino acids were highlighted for which mass shifts due to the interaction with sulfo-SDAD were indicated. These peptides were then analyzed using the Mass Matrix software. The sequences of peptides that were reproducibly identified in three independent experiments are presented.

## Supplementary Materials

Supplementary Materials can be found at https://www.mdpi.com/1422-0067/21/21/7843/s1. Supplementary Table: Table S1. List of peptide matches from the MassMatrix PC server. Supplementary Figures: Figure S1. Electrophoretic characteristics of purified fungal enolases. Figure S2. Homology model of *C. albicans* enolase based on the PDB structure: 1EBH (*S. cerevisiae* enolase). Figure S3. VTR structure obtained by using the software package Schrödinger Release 2019-1: Prime. Figure S4. Binding of *C. albicans* cell surface or cytosolic enolase to microplate-immobilized VTR, FN and HPG. Figure S5. Relative activity of enolase after its interaction with the human proteins. Figure S6. Competition between soluble unlabeled HPG and microplate-immobilized VTR for binding to biotinylated *C. albicans* enolase. Supplementary videos: Video S1. Proposed model of the interaction between *C. albicans* enolase (green) and human VTR (purple). Video S2. The interaction between *C. albicans* enolase and human FN—model 1. Video S3. The interaction between *C. albicans* enolase and human FN—model 2. Video S4. A model of the *C. albicans* enolase interaction with human HPG.

## Abbreviations

ECM	extracellular matrix
FN	fibronectin
FN-Bt	biotin-labeled fibronectin
HPG	human plasminogen
HPG-Bt	biotin-labeled human plasminogen
LC-MS/MS	liquid chromatography-coupled tandem mass spectrometry
R-Eno	recombinant enolase of *C. albicans*
R-Eno_sc_	recombinant enolase of *C. albicans* with a sequence of *S. cerevisiae*
SA-HRP/TMB	streptavidin-horseradish peroxidase/tetramethylbenzidine
SDS-PAGE	sodium dodecyl sulphate polyacrylamide gel electrophoresis
sulfo-SDAD	sulfosuccinimidyl 2-([4,4′-azipentanamido]ethyl)-1,3′-dithiopropionate
SPR	surface plasmon resonance
VTR	vitronectin
VTR-Bt	biotin-labeled vitronectin

## References

[1] Díaz-Ramos A., Roig-Borrellas A., García-Melero A., López-Alemany R. (2012). α-Enolase, a multifunctional protein: Its role on pathophysiological situations. J. Biomed. Biotechnol..

[2] Ji H., Wang J., Guo J., Li Y., Lian S., Guo W., Yang H., Kong F., Zhen L., Guo L. (2016). Progress in the biological function of alpha-enolase. Anim. Nutr..

[3] Didiasova M., Schaefer L., Wygrecka M. (2019). When place matters: Shuttling of enolase-1 across cellular compartments. Front. Cell Dev. Biol..

[4] Wistow G., Piatigorsky J. (1987). Recruitment of enzymes as lens structural proteins. Science.

[5] Wistow G.J., Lietman T., Williams L.A., Stapel S.O., de Jong W.W., Horwitz J., Piatigorsky J. (1988). τ-Crystallin/α-enolase: One gene encodes both an enzyme and a lens structural protein. J. Cell Biol..

[6] Jeffery C.J. (1999). Moonlighting proteins. Trends Biochem. Sci..

[7] Satala D., Karkowska-Kuleta J., Zelazna A., Rapala-Kozik M., Kozik A. (2020). Moonlighting proteins at the candidal cell surface. Microorganisms.

[8] Chandran V., Luisi B.F. (2006). Recognition of enolase in the *Escherichia coli* RNA degradosome. J. Mol. Biol..

[9] Morita T., Kawamoto H., Mizota T., Inada T., Aiba H. (2004). Enolase in the RNA degradosome plays a crucial role in the rapid decay of glucose transporter mRNA in the response to phosphosugar stress in *Escherichia coli*. Mol. Microbiol..

[10] Iida H., Yahara I. (1985). Yeast heat-shock protein of Mr 48,000 is an isoprotein of enolase. Nature.

[11] Brandina I., Graham J., Lemaitre-Guillier C., Entelis N., Krasheninnikov I., Sweetlove L., Tarassov I., Martin R.P. (2006). Enolase takes part in a macromolecular complex associated to mitochondria in yeast. Biochim. Biophys. Acta.

[12] Entelis N., Brandina I., Kamenski P., Krasheninnikov I.A., Martin R.P., Tarassov I. (2006). A glycolytic enzyme, enolase, is recruited as a cofactor of tRNA targeting toward mitochondria in *Saccharomyces cerevisiae*. Genes Dev..

[13] Keller A., Peltzer J., Carpentier G., Horváth I., Oláh J., Duchesnay A., Orosz F., Ovádi J. (2007). Interactions of enolase isoforms with tubulin and microtubules during myogenesis. Biochim. Biophys. Acta.

[14] Bottalico L.A., Kendrick N.C., Keller A., Li Y., Tabas I. (1993). Cholesteryl ester loading of mouse peritoneal macrophages is associated with changes in the expression or modification of specific cellular proteins, including increase in an α-enolase isoform. Arterioscler. Thromb..

[15] Shand J.H., West D.W. (1995). Inhibition of neutral cholesteryl ester hydrolase by the glycolytic enzyme enolase. Is this a secondary function of enolase?. Lipids.

[16] Graven K.K., Zimmerman L.H., Dickson E.W., Weinhouse G.L., Farber H.W. (1993). Endothelial cell hypoxia associated proteins are cell and stress specific. J. Cell. Physiol..

[17] Aaronson R.M., Graven K.K., Tucci M., McDonald R.J., Farber H.W. (1995). Non-Neuronal enolase is an endothelial hypoxic stress protein. J. Biol. Chem..

[18] Feo S., Arcuri D., Piddini E., Passantino R., Giallongo A. (2000). *ENO1* gene product binds to the c-myc promoter and acts as a transcriptional repressor: Relationship with Myc promoter-binding protein 1 (MBP-1). FEBS Lett..

[19] Lung J., Liu K.J., Chang J.Y., Leu S.J., Shih N.Y. (2010). MBP-1 is efficiently encoded by an alternative transcript of the *ENO1* gene but post-translationally regulated by proteasome-dependent protein turnover. FEBS J..

[20] Cappello P., Principe M., Bulfamante S., Novelli F. (2017). α-Enolase (*ENO1*), a potential target in novel immunotherapies. Front. Biosci..

[21] Wang G., Xia Y., Cui J., Gu Z., Song Y., Chen Y.Q., Chen H., Zhang H., Chen W. (2014). The roles of moonlighting proteins in bacteria. Curr. Issues Mol. Biol..

[22] Jeffery C.J. (2018). Intracellular proteins moonlighting as bacterial adhesion factors. AIMS Microbiol..

[23] Karkowska-Kuleta J., Kozik A. (2014). Moonlighting proteins as virulence factors of pathogenic fungi, parasitic protozoa and multicellular parasites. Mol. Oral Microbiol..

[24] Gómez-Arreaza A., Acosta H., Quiñones W., Concepción J.L., Michels P.A.M., Avilán L. (2014). Extracellular functions of glycolytic enzymes of parasites: Unpredicted use of ancient proteins. Mol. Biochem. Parasitol..

[25] López-Villar E., Monteoliva L., Larsen M.R., Sachon E., Shabaz M., Pardo M., Pla J., Gil C., Roepstorff P., Nombela C. (2006). Genetic and proteomic evidences support the localization of yeast enolase in the cell surface. Proteomics.

[26] Castillo L., Calvo E., Martínez A.I., Ruiz-Herrera J., Valentín E., Lopez J.A., Sentandreu R. (2008). A study of the *Candida albicans* cell wall proteome. Proteomics.

[27] Karkowska-Kuleta J., Zajac D., Bochenska O., Kozik A. (2015). Surfaceome of pathogenic yeasts, *Candida parapsilosis* and *Candida tropicalis*, revealed with the use of cell surface shaving method and shotgun proteomic approach. Acta Biochim. Pol..

[28] Karkowska-Kuleta J., Satala D., Bochenska O., Rapala-Kozik M., Kozik A. (2019). Moonlighting proteins are variably exposed at the cell surfaces of *Candida glabrata*, *Candida parapsilosis* and *Candida tropicalis* under certain growth conditions. BMC Microbiol..

[29] Mohamed A.A., Lu X.L., Mounmin F.A. (2019). Diagnosis and treatment of esophageal candidiasis: Current updates. Can. J. Gastroenterol. Hepatol..

[30] Tadec L., Talarmin J.P., Gastinne T., Bretonnière C., Miegeville M., Le Pape P., Morio F. (2016). Epidemiology, risk factor, species distribution, antifungal resistance and outcome of candidemia at a single French hospital: A 7 year study. Mycoses.

[31] Enoch D.A., Yang H., Aliyu S.H., Micallef C. (2017). The changing epidemiology of invasive fungal infections. Methods Mol. Biol..

[32] Lamoth F., Lockhart S.R., Berkow E.L., Calandra T. (2018). Changes in the epidemiological landscape of invasive candidiasis. J. Antimicrob. Chemother..

[33] Giri S., Kindo A.J. (2012). A review of *Candida* species causing blood stream infection. Indian J. Med. Microbiol..

[34] Wu P.F., Liu W.L., Hsieh M.H., Hii I.M., Lee Y.L., Lin Y.T., Ho M.-W., Liu C.-E., Chen Y.-H., Wang F.-D. (2017). Epidemiology and antifungal susceptibility of candidemia isolates of non-albicans *Candida* species from cancer patients. Emerg. Microbes Infect..

[35] Sundstrom P., Aliaga G.R. (1992). Molecular cloning of cDNA and analysis of protein secondary structure of *Candida albicans* enolase, an abundant, immunodominant glycolytic enzyme. J. Bacteriol..

[36] Ko H.C., Hsiao T.Y., Chen C.T., Yang Y.L. (2013). *Candida albicans ENO1* null mutants exhibit altered drug susceptibility, hyphal formation, and virulence. J. Microbiol..

[37] Reyna-Beltrán E., Iranzo M., Calderón-González K.G., Mondragón-Flores R., Labra-Barrios M.L., Mormeneo S., Luna-Arias J.P. (2018). The *Candida albicans ENO1* gene encodes a transglutaminase involved in growth, cell division, morphogenesis, and osmotic protection. J. Biol. Chem..

[38] Núñez-Beltrán A., López-Romero E., Cuéllar-Cruz M. (2017). Identification of proteins involved in the adhesion of *Candida* species to different medical devices. Microb. Pathog..

[39] Silva S., Negri M., Henriques M., Oliveira R., Williams D.W., Azeredo J. (2011). Adherence and biofilm formation of non-Candida albicans *Candida* species. Trends Microbiol..

[40] Silva R.C., Padovan A.C., Pimenta D.C., Ferreira R.C., da Silva C.V., Briones M.R. (2014). Extracellular enolase of *Candida albicans* is involved in colonization of mammalian intestinal epithelium. Front. Cell. Infect. Microbiol..

[41] Ramírez-Quijas M.D., López-Romero E., Cuéllar-Cruz M. (2015). Proteomic analysis of cell wall in four pathogenic species of *Candida* exposed to oxidative stress. Microb. Pathog..

[42] Gil-Bona A., Amador-García A., Gil C., Monteoliva L. (2018). The external face of *Candida albicans*: A proteomic view of the cell surface and the extracellular environment. J. Proteom..

[43] Wächtler B., Citiulo F., Jablonowski N., Förster S., Dalle F., Schaller M., Wilson D., Hube B. (2012). Candida albicans-epithelial interactions: Dissecting the roles of active penetration, induced endocytosis and host factors on the infection process. PLoS ONE.

[44] Felk A., Kretschmar M., Albrecht A., Schaller M., Beinhauer S., Nichterlein T., Sanglard D., Korting H.C., Schäfer W., Hube B. (2002). *Candida albicans* hyphal formation and the expression of the Efg1-regulated proteinases Sap4 to Sap6 are required for the invasion of parenchymal organs. Infect. Immun..

[45] Kozik A., Karkowska-Kuleta J., Zajac D., Bochenska O., Kedracka-Krok S., Jankowska U., Rapala-Kozik M. (2015). Fibronectin-, vitronectin- and laminin-binding proteins at the cell walls of *Candida parapsilosis* and *Candida tropicalis* pathogenic yeasts. BMC Microbiol..

[46] Kinloch A., Tatzer V., Wait R., Peston D., Lundberg K., Donatien P., Moyes D.L., Taylor P.C., Venables P.J. (2005). Identification of citrullinated alpha-enolase as a candidate autoantigen in rheumatoid arthritis. Arthritis Res. Ther..

[47] Agarwal A.K., Xu T., Jacob M.R., Feng Q., Li X.C., Walker L.A., Clark A.M. (2008). Genomic and genetic approaches for the identification of antifungal drug targets. Infect. Disord. Drug Targets.

[48] Poltermann S., Kunert A., von der Heide M., Eck R., Hartmann A., Zipfel P.F. (2007). Gpm1p is a factor H-, FHL-1-, and plasminogen-binding surface protein of *Candida albicans*. J. Biol. Chem..

[49] Karkowska-Kuleta J., Zajac D., Bras G., Bochenska O., Rapala-Kozik M., Kozik A. (2017). Binding of human plasminogen and high-molecular-mass kininogen by cell surface-exposed proteins of *Candida parapsilosis*. Acta Biochim. Pol..

[50] Crowe J.D., Sievwright I.K., Auld G.C., Moore N.R., Gow N.A., Booth N.A. (2003). *Candida albicans* binds human plasminogen: Identification of eight plasminogen-binding proteins. Mol. Microbiol..

[51] Funk J., Schaarschmidt B., Slesiona S., Hallström T., Horn U., Brock M. (2016). The glycolytic enzyme enolase represents a plasminogen-binding protein on the surface of a wide variety of medically important fungal species. Int. J. Med. Microbiol..

[52] Jeffery C.J. (2005). Mass spectrometry and the search for moonlighting proteins. Mass Spectrom. Rev..

[53] Jeffery C.J. (2016). Protein species and moonlighting proteins: Very small changes in a protein’s covalent structure can change its biochemical function. J. Proteom..

[54] Hernández S., Franco L., Calvo A., Ferragut C., Hermoso A., Amela I., Gómez A., Querol E., Cedano J. (2015). Bioinformatics and moonlighting proteins. Front. Bioeng. Biotechnol..

[55] Ehinger S., Schubert W.D., Bergmann S., Hammerschmidt S., Heinz D.W. (2004). Plasmin(ogen)-binding a-enolase from *Streptococcus pneumoniae*: Crystal structure and evaluation of plasmin(ogen)-binding sites. J. Mol. Biol..

[56] Kang H.J., Jung S.K., Kim S.J., Chung S.J. (2008). Structure of human α-enolase (hENO1), a multifunctional glycolytic enzyme. Acta Crystallogr..

[57] Godier A., Hunt B.J. (2013). Plasminogen receptors and their role in the pathogenesis of inflammatory, autoimmune and malignant disease. J. Thromb. Haemost..

[58] Wedekind J.E., Reed G.H., Rayment I. (1995). Octahedral coordination at the high-affinity metal site in enolase: Crystallographic analysis of the MgII-enzyme complex from yeast at 1.9 A resolution. Biochemistry.

[59] Law R.H., Caradoc-Davies T., Cowieson N., Horvath A.J., Quek A.J., Encarnacao J.A., Steer D., Cowan A., Zhang Q., Lu B.G. (2012). The X-ray crystal structure of full-length human plasminogen. Cell Rep..

[60] Graille M., Pagano M., Rose T., Ravaux M.R., van Tilbeurgh H. (2010). Zinc induces structural reorganization of gelatin binding domain from human fibronectin and affects collagen binding. Structure.

[61] Tochowicz A., Goettig P., Evans R., Visse R., Shitomi Y., Palmisano R.G., Ito N., Richter K., Maskos K., Franke D. (2011). The dimer interface of the membrane type 1 matrix metalloproteinase hemopexin domain: Crystal structure and biological functions. J. Biol. Chem..

[62] Zhou A., Huntington J.A., Pannu N.S., Carrell R.W., Read R.J. (2003). How vitronectin binds PAI-1 to modulate fibrinolysis and cell migration. Nat. Struct. Biol..

[63] Kjaergaard M., Gårdsvoll H., Hirschberg D., Nielbo S., Mayasundari A., Peterson C.B., Jansson A., Jørgensen T.J., Poulsen F.M., Ploug M. (2007). Solution structure of recombinant somatomedin B domain from vitronectin produced in *Pichia pastoris*. Protein Sci..

[64] Huai Q., Zhou A., Lin L., Mazar A.P., Parry G.C., Callahan J., Shaw D.E., Furie B., Furie B.C., Huang M. (2008). Crystal structures of two human vitronectin, urokinase and urokinase receptor complexes. Nat. Struct. Mol. Biol..

[65] Jeffery C.J. (2019). Multitalented actors inside and outside the cell: Recent discoveries add to the number of moonlighting proteins. Biochem. Soc. Trans..

[66] Lee P.Y., Gam L.H., Yong V.C., Rosli R., Ng K.P., Chong P.P. (2014). Identification of immunogenic proteins of *Candida parapsilosis* by serological proteome analysis. J. Appl. Microbiol..

[67] Lee P.Y., Gam L.H., Yong V.C., Rosli R., Ng K.P., Chong P.P. (2014). Immunoproteomic analysis of antibody response to cell wall-associated proteins of *Candida tropicalis*. J. Appl. Microbiol..

[68] Karkowska-Kuleta J., Zajac D., Bras G., Bochenska O., Seweryn K., Kędracka-Krok S., Jankowska U., Rapala-Kozik M., Kozik A. (2016). Characterization of the interactions between human high-molecular-mass kininogen and cell wall proteins of pathogenic yeasts *Candida tropicalis*. Acta Biochim. Pol..

[69] Karkowska-Kuleta J., Kulig K., Karnas E., Zuba-Surma E., Woznicka O., Pyza E., Kuleta P., Osyczka A., Rapala-Kozik M., Kozik A. (2020). Characteristics of extracellular vesicles released by the pathogenic yeast-like fungi *Candida glabrata*, *Candida parapsilosis* and *Candida tropicalis*. Cells.

[70] Dallo S.F., Kannan T.R., Blaylock M.W., Baseman J.B. (2002). Elongation factor Tu and E1 βsubunit of pyruvate dehydrogenase complex act as fibronectin binding proteins in *Mycoplasma pneumoniae*. Mol. Microbiol..

[71] Heilmann C., Hartleib J., Hussain M.S., Peters G. (2005). The multifunctional *Staphylococcus aureus* autolysin aaa mediates adherence to immobilized fibrinogen and fibronectin. Infect. Immun..

[72] Kinhikar A.G., Vargas D., Li H., Mahaffey S.B., Hinds L., Belisle J.T., Laal S. (2006). Mycobacterium tuberculosis malate synthase is a laminin-binding adhesin. Mol. Microbiol..

[73] Esgleas M., Li Y.Y., Hancock M.A., Hare J., Dubreui J.D., Gottschalk M. (2008). Isolation and characterization of α-enolase, a novel fibronectin-binding protein from *Streptococcus suis*. Microbiology.

[74] Dasari P., Koleci N., Shopova I.A., Wartenberg D., Beyersdorf N., Dietrich S., Sahagún-Ruiz A., Figge M.T., Skerka C., Brakhage A.A. (2019). Enolase from *Aspergillus fumigatus* is a moonlighting protein that binds the human plasma complement proteins factor H, FHL-1, C4BP, and plasminogen. Front. Immunol..

[75] Seweryn K., Karkowska-Kuleta J., Wolak N., Bochenska O., Kedracka-Krok S., Kozik A., Rapala-Kozik M. (2015). Kinetic and thermodynamic characterization of the interactions between the components of human plasma kinin-forming system and isolated and purified cell wall proteins of *Candida albicans*. Acta Biochim. Pol..

[76] Donohue D.S., Ielasi F.S., Goossens K.V., Willaert R.G. (2011). The N-terminal part of Als1 protein from *Candida albicans* specifically binds fucose-containing glycans. Mol. Microbiol..

[77] Ielasi F.S., Verhaeghe T., Desmet T., Willaert R.G. (2014). Engineering the carbohydrate-binding site of Epa1p from *Candida glabrata*: Generation of adhesin mutants with different carbohydrate specificity. Glycobiology.

[78] Zajac D., Karkowska-Kuleta J., Bochenska O., Rapala-Kozik M., Kozik A. (2016). Interaction of human fibronectin with *Candida glabrata* epithelial adhesin 6 (Epa6). Acta Biochim. Pol..

[79] Butterfield D.A., Lange M.L. (2009). Multifunctional roles of enolase in Alzheimer’s disease brain: Beyond altered glucose metabolism. J. Neurochem..

[80] Zakrzewicz D., Didiasova M., Krüger M., Giaimo B.D., Borggrefe T., Mieth M., Hocke A.C., Zakrzewicz A., Schaefer L., Preissner K.T. (2018). Protein arginine methyltransferase 5 mediates enolase-1 cell surface trafficking in human lung adenocarcinoma cells. Biochim. Biophys. Acta Mol. Basis Dis..

[81] Shevade S., Jindal N., Dutta S., Jarori G.K. (2013). Food vacuole associated enolase in plasmodium undergoes multiple post-translational modifications: Evidence for atypical ubiquitination. PLoS ONE.

[82] Zhang E., Brewer J.M., Minor W., Carreira L.A., Lebioda L. (1997). Mechanism of enolase: The crystal structure of asymmetric dimer enolase-2-phospho-D-glycerate/enolase-phosphoenolpyruvate at 2.0 Å resolution. Biochemistry.

[83] Piccard H., Van den Steen P.E., Opdenakker G. (2007). Hemopexin domains as multifunctional liganding modules in matrix metalloproteinases and other proteins. J. Leukoc. Biol..

[84] Fox D., Smulian A.G. (2001). Plasminogen-binding activity of enolase in the opportunistic pathogen *Pneumocystis carinii*. Med. Mycol..

[85] Marcos C.M., Silva J.F., Oliveira H.C., Silva R.A.M., Mendes-Giannini M.J.S., Fusco-Almeida A.M. (2012). Surface-Expressed enolase contributes to the adhesion of *Paracoccidioides brasiliensis* to host cells. FEMS Yeast Res..

[86] Ceremuga I., Seweryn E., Bednarz-Misa I., Pietkiewicz J., Jermakow K., Banas T., Gamian A. (2014). Enolase-like protein present on the outer membrane of *Pseudomonas aeruginosa* binds plasminogen. Folia Microbiol..

[87] Bao S., Guo X., Yu S., Ding J., Tan L., Zhang F., Sun Y., Qiu X., Chen G., Ding C. (2014). *Mycoplasma synoviae* enolase is a plasminogen/fibronectin binding protein. BMC Vet. Res..

[88] Derbise A., Song Y.P., Parikh S., Fischetti V.A., Pancholi V. (2004). Role of the C-terminal lysine residues of streptococcal surface enolase in Glu- and Lys-plasminogen-binding activities of group A streptococci. Infect. Immun..

[89] Sha J., Erova T.E., Alyea R.A., Wang S., Olano J.P., Pancholi V., Chopra A.K. (2009). Surface-Expressed enolase contributes to the pathogenesis of clinical isolate SSU of *Aeromonas hydrophila*. J. Bacteriol..

[90] Rahi A., Dhiman A., Singh D., Lynn A.M., Rehan M., Bhatnagar R. (2017). Exploring the interaction between *Mycobacterium tuberculosis* enolase and human plasminogen using computational methods and experimental techniques. J. Cell. Biochem..

[91] Bergmann S., Wild D., Diekmann O., Frank R., Bracht D., Chhatwal G.S., Hammerschmidt S. (2003). Identification of a novel plasmin(ogen)-binding motif in surface displayed alpha-enolase of *Streptococcus pneumoniae*. Mol. Microbiol..

[92] Vanegas G., Quiñones W., Carrasco-López C., Concepción J.L., Albericio F., Avilán L. (2007). Enolase as a plasminogen binding protein in *Leishmania mexicana*. Parasitol. Res..

[93] Nogueira S.V., Fonseca F.L., Rodrigues M.L., Mundodi V., Abi-Chacra E.A., Winters M.S., Alderete J.F., de Almeida Soares C.M. (2010). *Paracoccidioides brasiliensis* enolase is a surface protein that binds plasminogen and mediates interaction of yeast forms with host cells. Infect. Immun..

[94] Ghosh A.K., Jacobs-Lorena M. (2011). Surface-expressed enolases of *Plasmodium* and other pathogens. Mem. Inst. Oswaldo Cruz.

[95] López-López M.J., Rodríguez-Luna I.C., Lara-Ramírez E.E., López-Hidalgo M., Benítez-Cardoza C.G., Guo X. (2018). Biochemical and biophysical characterization of the enolase from *Helicobacter pylori*. Biomed. Res. Int..

[96] Rios-Steiner J.L., Schenone M., Mochalkin I., Tulinsky A., Castellino F.J. (2001). Structure and binding determinants of the recombinant kringle-2 domain of human plasminogen to an internal peptide from a group A *Streptococcal* surface protein. J. Mol. Biol..

[97] Bhattacharya S., Ploplis V.A., Castellino F.J. (2012). Bacterial plasminogen receptors utilize host plasminogen system for effective invasion and dissemination. J. Biomed. Biotechnol..

[98] Vieira M.L., Vasconcellos S.A., Goncales A.P., de Morais Z.M., Nascimento A.L. (2009). Plasminogen acquisition and activation at the surface of *Leptospira* species lead to fibronectin degradation. Infect. Immun..

[99] Ballantyne D.S., Warmington J.R. (2000). Purification of native enolase from medically important *Candida* species. Biotechnol. Appl. Biochem..

[100] Rapala-Kozik M., Karkowska J., Jacher A., Golda A., Barbasz A., Guevara-Lora I., Kozik A. (2008). Kininogen adsorption to the cell surface of *Candida* spp.. Int. Immunopharmacol..

[101] Xu H., Freitas M.A. (2009). MassMatrix: A database search program for rapid characterization of proteins and peptides from tandem mass spectrometry data. Proteomics.

[102] Madeira F., Park Y.M., Lee J., Buso N., Gur T., Madhusoodanan N., Basutkar P., Tivey A.R.N., Potter S.C., Finn R.D. (2019). The EMBL-EBI search and sequence analysis tools APIs in 2019. Nucleic Acids Res..

[103] Sali A., Blundell T.L. (1993). Comparative protein modelling by satisfaction of spatial restraints. J. Mol. Biol..

[104] Vajda S., Yueh C., Beglov D., Bohnuud T., Mottarella S.E., Xia B., Hall D.R., Kozakov D. (2017). New additions to the ClusPro server motivated by CAPRI. Proteins.

[105] Kozakov D., Hall D.R., Xia B., Porter K.A., Padhorny D., Yueh C., Beglov D., Vajda S. (2017). The ClusPro web server for protein-protein docking. Nat. Protoc..

