# Supplementary material for: Structural Insights into the Interactions of Candidal Enolase with Human Vitronectin, Fibronectin and Plasminogen
Source: Int J Mol Sci. 2020 Oct 22;21(21):7843. doi: 10.3390/ijms21217843 (PMC7660097; doi:10.3390/ijms21217843)
Supplement: Supplementary file 1 [file ijms-21-07843-s001.zip › Supplementary Materials/Supplementary materials.pdf]

SUPPLEMENTARY TABLE

Table S1. List of peptide matches from the MassMatrix PC server.

Peptide	Charge	Score	pp value	pp2 value
***C. albicans* enolase**				
KAGYKGKVGIAMD	+2	88	16.6	15.8
VGIAMDVASSEFYKDGKYDLDFK	+2	113	22.8	14.4
NPTRIKTAIEK	+2	46	20.7	8.4
IEEELGSEAIYAGK	+3	83	8.0	12.8
DGDKSK	+2	41	8.7	7.0
LGANAILGVSLAAANAAAAAQGIPLYKHIANISNAKK	+5	51	7.5	7.4
IGSEVYHNLK	+2	60	18.1	17.4
KGKVGIAMDVASSEFYKDGK	+4	24	9.0	9.8
GLFRSIVPSGASTGVHEALELRDGDKS	+2	37	18.0	7.3
SKWLGKGVLK	+3	36	10.6	7.9
LGANAILGVSLAAANAAAAAQGIPLYK	+2	54	18.9	12.3
AGYKGKVGIAMDVASSEFYKDGK	+2	49	21.2	8.2
***C. tropicalis* enolase**				
QAGHTGKVKIAMDPASSE	+2	68	13.7	8.9
IAMDPASSEFFKDGKYDLDFK	+2	46	10.8	9.0
IKKAIEKK	+2	42	8.1	9.6
IAMDPASSEFFKDGKYDLDFK	+2	67	11.9	12.7
KLGANAILGVSLAAA	+2	43	7.3	8.2
TPEEALDLIVESIEQAGHTGK	+3	68	16.7	17.7
IAMDPASSEFFKDGK	+2	67	11.9	12.7
**VTR**				
DVWGIEGPIDAAFTR	+2	53	12.2	9.0
**HPG**				
ECAAKCEE	+2	77	25.6	12.0
TNPRAGLE	+2	44	11.0	7.6

SUPPLEMENTARY FIGURES

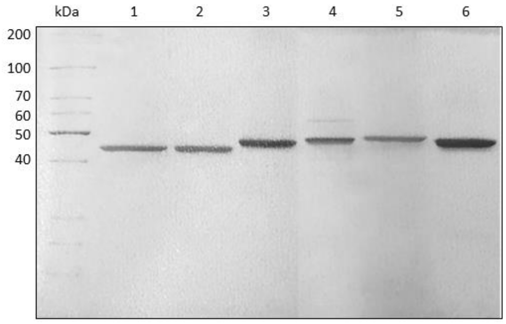

Fig. S1. Electrophoretic characteristics of purified fungal enolases: (1) cell-surface enolase of *C. albicans*; (2) cytosolic enolase of *C. albicans*; (3) cytosolic enolase of *C. tropicalis*; (4) commercial enolase of *S. cerevisiae*; (5) recombinant enolase of *C. albicans* (R-Eno); (6) recombinant enolase of *C. albicans* with the sequence D_{235}-Y_{255} substituted with the sequence K_{234}-F_{254} of *S. cerevisiae* enolase (R-Eno_{sc}). Purified enolases were separated by SDS-PAGE under reducing conditions with the Laemmli system using a 12% separating gel, and then visualized by silver staining. The molecular-mass marker bands are indicated in the leftmost lane.

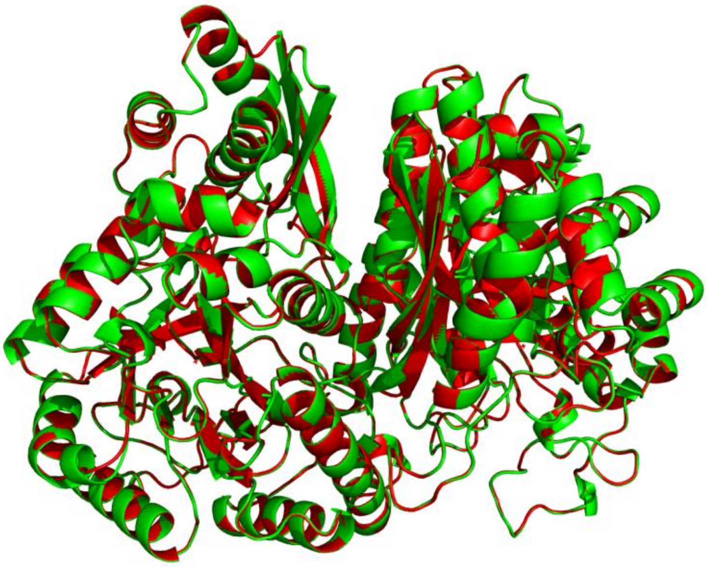

Fig. S2. Homology model of *C. albicans* enolase based on the PDB structure: 1EBH (*S. cerevisiae* enolase) [58]. The model and template are denoted in red and green, respectively.

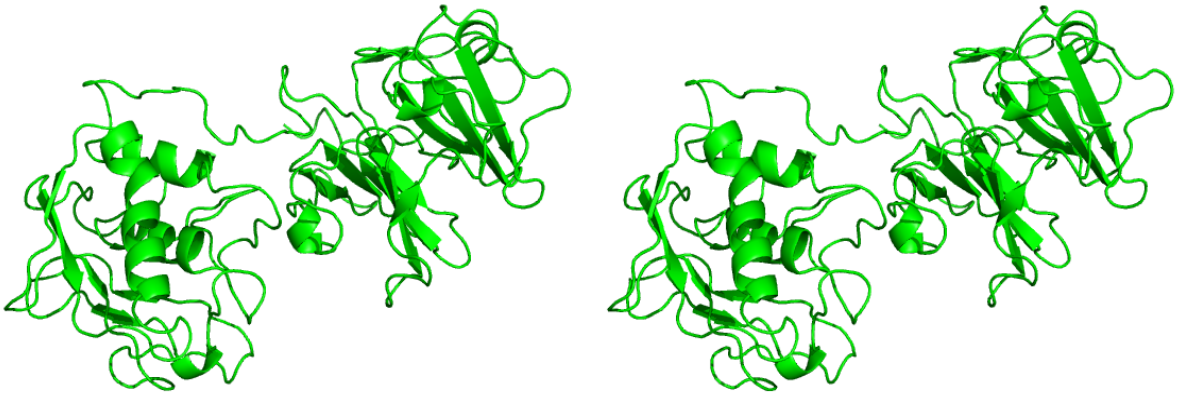

Fig. S3. VTR structure obtained by using the software package Schrödinger Release 2019-1: Prime based on multiple templates (PDB ID: 3C7X, 1OC0, 2JQ8 and 3BT1 [61-64]). The 3D molecular model is presented in a wall-eyed stereo view.

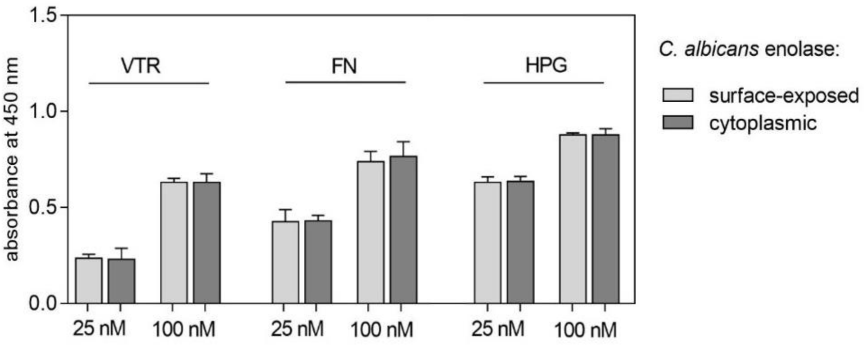

Fig. S4. Binding of *C. albicans* cell surface or cytosolic enolase to microplate-immobilized VTR, FN and HPG.

Microplate wells coated with 3 pmoles of VTR, FN or HPG were filled with 50 µl of biotin-labeled *C. albicans* enolases at increasing concentrations. The results obtained during the control ("no human protein") experiment were subtracted from the total binding. The bars represent the mean values ± standard deviation (3 determinations).

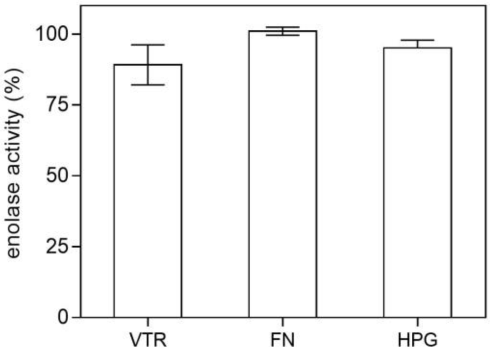

Fig. S5. Relative activity of enolase after its interaction with the human proteins. Enolase (20 nM) was incubated with VTR, FN and HPG at a 1:1 molar ratio for 1 h in PBS at 37°C, with gentle shaking. The enolase activity was then monitored for 1 h. The results obtained during the control experiment ("no human protein") were taken to indicate 100% enolase activity. The bars represent the mean values ± standard deviation (3 determinations).

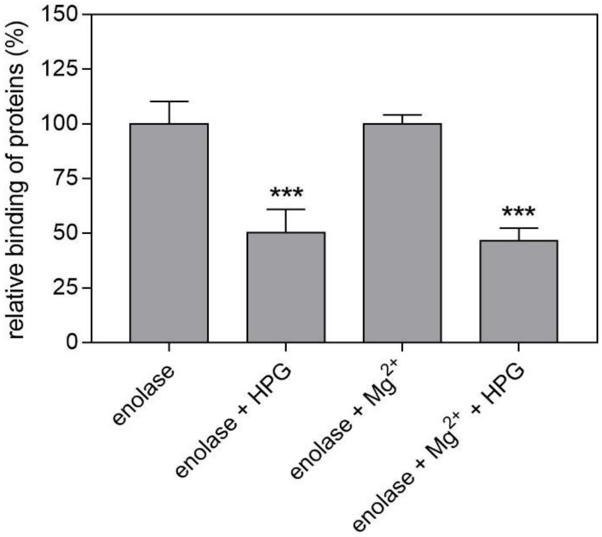

Fig. S6. Competition between soluble unlabeled HPG and microplate-immobilized VTR for binding to biotinylated *C. albicans* enolase.

A solution of 20 nM biotinylated *C. albicans* enolase (50 µl) or a mixture of 40 nM solution of biotinylated enolase (25 µl) and 400 nM HPG (25 µl) in PBS was added to the microplate well with immobilized VTR (5 pmol). After incubation for 1.5 h at 37°C, the amount of bound biotinylated enolase was determined using the SA-HRP/TMB detection system. In a variation of this experiment, Mg^{2+} at 5 mM was additionally present in the protein solutions in PBS. The wells without HPG served as a 100% reference control. Bars on the graph represent the mean values from 3 determinations ± standard deviation. Statistical significance levels against the controls (wells without HPG added) were determined with a one-way ANOVA test; ***$p < 0.001$.

SUPPLEMENTARY VIDEO LEGENDS

Video S1. Proposed model of the interaction between *C. albicans* enolase (green) and human VTR (purple).

The enolase peptides and VTR peptide identified in the chemical mapping experiments are indicated in red and gray, respectively. The active site residues of the catalytic center of enolase are indicated in yellow.

Video S2. The interaction between *C. albicans* enolase and human FN – model 1.

The model for the protein-protein interaction between a human FN fragment (pink) and *C. albicans* enolase (green with active site residues of the catalytic center indicated in yellow) were generated using ClusPro 2.0: protein-protein docking software. The enolase peptides identified in the chemical mapping experiments are marked in red.

Video S3. The interaction between *C. albicans* enolase and human FN – model 2.

The models for the protein-protein interaction between a human FN fragment (black) and *C. albicans* enolase (green with active site residues of the catalytic center indicated in yellow) were generated using ClusPro 2.0: protein-protein docking software. The enolase peptides identified in the chemical mapping experiments are marked in red.

Video S4. A model of the *C. albicans* enolase interaction with human HPG.

The enolase dimer is indicated in green and HPG is denoted in blue. The enolase and HPG peptides identified in the chemical mapping experiments are marked in red and gray, respectively. The active site residues of the catalytic center of the enolase are highlighted in yellow.
